# Multiplexed pancreatic genome engineering and cancer induction by transfection-based CRISPR/Cas9 delivery in mice

**DOI:** 10.1038/ncomms10770

**Published:** 2016-02-26

**Authors:** Roman Maresch, Sebastian Mueller, Christian Veltkamp, Rupert Öllinger, Mathias Friedrich, Irina Heid, Katja Steiger, Julia Weber, Thomas Engleitner, Maxim Barenboim, Sabine Klein, Sandra Louzada, Ruby Banerjee, Alexander Strong, Teresa Stauber, Nina Gross, Ulf Geumann, Sebastian Lange, Marc Ringelhan, Ignacio Varela, Kristian Unger, Fengtang Yang, Roland M. Schmid, George S. Vassiliou, Rickmer Braren, Günter Schneider, Mathias Heikenwalder, Allan Bradley, Dieter Saur, Roland Rad

**Affiliations:** 1 Department of Medicine II, Klinikum rechts der Isar, Technische Universität München, 81675 Munich, Germany; 2 German Cancer Consortium (DKTK), German Cancer Research Center (DKFZ), 69120 Heidelberg, Germany; 3 Wellcome Trust Sanger Institute, Wellcome Trust Genome Campus, Hinxton, Cambridge CB10 1SA, UK; 4 Institute of Radiology, Klinikum rechts der Isar, Technischen Universität München, 81675 Munich, Germany; 5 Department of Pathology, Klinikum rechts der Isar, Technische Universität München, 81675 Munich, Germany; 6 Institute of Virology, Technische Universität München/Helmholtz Zentrum München, 81675 Munich, Germany; 7 Instituto de Biomedicina y Biotecnología de Cantabria, 39011 Santander, Spain; 8 Helmholtz Zentrum München, Research Unit Radiation Cytogenetics, 85764 Neuherberg, Germany; 9 Division of Chronic Inflammation and Cancer, German Cancer Research Center (DKFZ), 69120 Heidelberg, Germany

## Abstract

Mouse transgenesis has provided fundamental insights into pancreatic cancer, but is limited by the long duration of allele/model generation. Here we show transfection-based multiplexed delivery of CRISPR/Cas9 to the pancreas of adult mice, allowing simultaneous editing of multiple gene sets in individual cells. We use the method to induce pancreatic cancer and exploit CRISPR/Cas9 mutational signatures for phylogenetic tracking of metastatic disease. Our results demonstrate that CRISPR/Cas9-multiplexing enables key applications, such as combinatorial gene-network analysis, *in vivo* synthetic lethality screening and chromosome engineering. Negative-selection screening in the pancreas using multiplexed-CRISPR/Cas9 confirms the vulnerability of pancreatic cells to *Brca2*-inactivation in a *Kras*-mutant context. We also demonstrate modelling of chromosomal deletions and targeted somatic engineering of inter-chromosomal translocations, offering multifaceted opportunities to study complex structural variation, a hallmark of pancreatic cancer. The low-frequency mosaic pattern of transfection-based CRISPR/Cas9 delivery faithfully recapitulates the stochastic nature of human tumorigenesis, supporting wide applicability for biological/preclinical research.

## Abstract (web summary)

CRISPR/Cas9 technology has been used for genome engineering *in vivo*. Here, the authors use a transfection technique to deliver multiple guide RNAs to the pancreas of adult mice, allowing genetic screening and chromosome engineering in pancreatic cancer.

## Graphical abstract

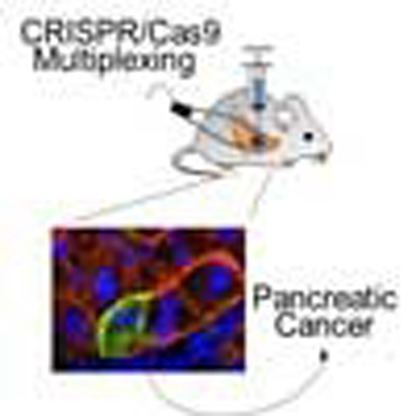

Pancreatic ductal adenocarcinoma (PDAC) has one of the most dismal prognoses of all cancer types. It is currently the fourth leading cause of cancer-related death worldwide and is expected to become the second within the next 20 years^1^. The therapeutic opportunities of advanced disease are very limited and five-year survival rates continued to remain at ∼5–7% in the past decades. Next-generation sequencing (NGS) of human PDAC and transposon-based genetic screening in mice have created large catalogues of genes involved in tumour development, but the complexity of the molecular processes driving the disease is still far from being understood^2^,^3^,^4^,^5^,^6^,^7^. A major challenge will be to assign biological relevance and molecular function to these large gene sets and to understand how complex genetic interactions drive the pathogenetic process. Likewise, pinpointing drivers among the thousands of transcriptionally or epigenetically dysregulated genes in a cancer is complex and limited by the lack of tools for high-throughput functional cancer genome analyses.

The development of methodologies to genetically target the mouse germ line^8^,^9^ has opened tremendous opportunities for gene function analysis. Sophisticated mouse models of pancreatic cancer have given extensive insights into many fundamental aspects of tumorigenesis that can only be studied at an organismal level^10^. Bottlenecks and limitations of classic transgenesis are, however, (i) the long time frames needed to generate and intercross genetically modified mice, (ii) the difficulties to model some aspects of the human disease (for example, the stochastic nature of somatic mutations in adult mice), (iii) the lack of high-throughput methods for functional interrogation of complex genetic interactions, (iv) the confounding phenotypes emerging in multiallelic crosses of transgenic mice generated in various genetic backgrounds and (v) the lack of tools for efficient modelling of the complex structural variations defining human cancer.

The prokaryotic CRISPR/Cas9 system has recently emerged as a powerful tool for genome engineering in mammalian cells^11^,^12^,^13^,^14^,^15^,^16^,^17^. Using programmable 20-bp single-guide RNAs (sgRNAs), the endonuclease Cas9 can be directed to desired genomic positions to induce DNA double-strand breaks. These breaks are repaired by imperfect non-homologous end joining, which can be exploited to induce insertions or deletions (indels) for heterozygous or homozygous gene inactivation. CRISPR/Cas9-based manipulation of haematopoietic stem cells or cultured epithelial cells/organoids followed by transplantation has been performed *ex vivo*^18^,^19^,^20^,^21^. In addition, we and others showed recently CRISPR/Cas9-based somatic genome editing in different organs of mice, including the lung, liver, brain and pancreas^22^,^23^,^24^,^25^,^26^,^27^,^28^,^29^ and direct *in vivo* forward genetic screening^29^. However, most approaches to deliver CRISPR/Cas9 *in vivo* have limitations, such as the inability or low efficiency of vector multiplexing for complex combinatorial gene editing or the high risk to induce off-target effects due to continuous activity of the system, for example, by virally delivered stably integrated CRISPR/Cas9.

To address these and other unsolved limitations, we have developed an electroporation-based vector delivery approach for multiplexed transient CRISPR/Cas9 targeting of the pancreas. We show how this methodology can be exploited for combinatorial gene targeting, negative-selection screening and chromosome engineering in pancreatic cancer. This approach will facilitate high-throughput analysis of gene function, of cancer gene interactions and of structural variations. We also pinpoint limitations of high-level multiplexing, providing guidance for appropriate use of the method.

## Results

### Transfection-based DNA delivery to pancreatic cells

**Figure 1:**
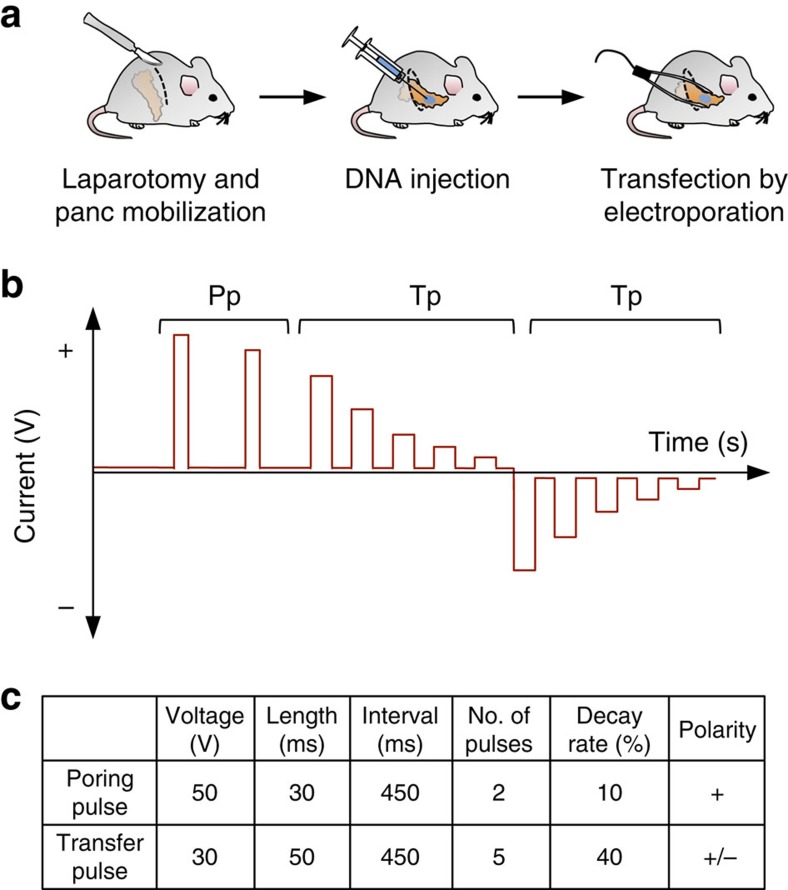
Electroporation-based plasmid delivery to the murine pancreas. (**a**) Scheme of experimental procedures. (**b**) Square-pulse generation for *in vivo* electroporation. Pp, poring pulses to induce pore formation in cellular membranes. Tp, reversed-phase transfer pulses (five prolonged peaks) for electro-kinetic transfer of DNA particles into cells. (**c**) Electroporation protocol used for optimized vector delivery into pancreatic (panc) cells.

In an attempt to co-deliver multiple CRISPR/Cas9 vectors to pancreatic cells, we have explored the possibility of targeting the pancreas by direct intra-pancreatic DNA injection and *in vivo* electroporation. After laparotomy, the pancreas can be mobilized to make it accessible for intraparenchymal injection of vector DNA. We instilled 50 μl of plasmid solution before applying an electric pulse for transfection. Settings of the Nepa21 square-pulse generator were optimized to efficiently target the local pancreatic parenchyma (Fig. 1 and ‘Methods' section).

**Figure 2:**
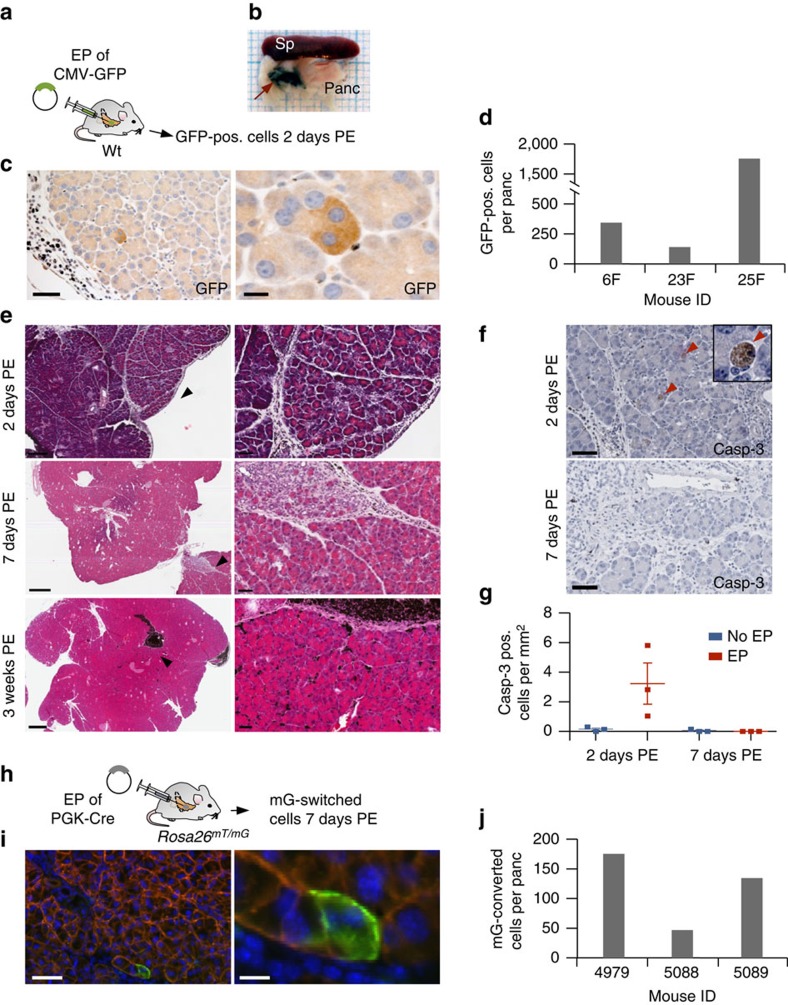
Efficiency of electroporation-based vector delivery into pancreatic cells. (**a**–**d**) Assessing vector delivery efficiency 2 days post electroporation (PE). (**a**) A GFP expression vector was injected into pancreata of wild-type mice. Two days PE, the mice were killed and pancreata analysed for transiently expressed GFP. (**b**) Endoscopic marker was used for marking of the electroporated area to allow its identification at necropsy (red arrow). Sp, spleen. Panc, pancreas. (**c**) IHC against GFP showing positive acinar cells at the site of electroporation. Note the low frequency of GFP-positive (pos.) cells. Scale bars, 50 μm (left), 10 μm (right). (**d**) Absolute numbers of GFP-positive cells in electroporated pancreata of indicated mice. (**e**) Pancreatic histopathology at 2, 7 and 21 days PE. Top panels: haematoxylin and eosin staining 2 days PE showing slight-to-moderate inter- and intralobular infiltration of macrophages and neutrophils at the site of electroporation. Few acinar cells show cytoplasmic vacuolization. Middle panels: moderate interlobular infiltration with macrophages, neutrophils and lymphocytes 7 days PE. Bottom panels: complete regeneration of the acinar cell compartment and disappearance of inflammatory response in a mouse 3 weeks PE. In all the pictures, dark pigment originating from the endoscopic marker is cleared by macrophages (black accumulations of cells). Black arrow heads indicate the magnified areas. Scale bars, top left 200 μm, mid/bottom left 500 μm and right column 50 μm. (**f**–**g**) IHC of caspase-3 for quantification of apoptosis 2 and 7 days PE. (**f**) Caspase-3-positive cells at the site of electroporation (top) and the surrounding normal pancreatic tissue (bottom). Arrow heads indicate apoptotic cells. Scale bars, 50 μm. (**g**) Enumeration of caspase-3-positive cells. Graph shows mean±s.e.m. (**h**–**j**) Assessing the number of long-term surviving cells 7 days PE. (**h**) A Cre recombinase expression vector was electroporated into pancreata of *Rosa26*^*mT/mG*^ mice to induce recombination of the *Rosa26*^*mT/mG*^ reporter allele. (**i**) Conversion of membranous red to cytoplasmic/membranous green fluorescence in acinar cells of electroporated pancreata. Scale bars, 50 μm (left) and 10 μm (right). Note the low number of cells with mT/mG conversion. (**j**) Absolute numbers of green fluorescent cells in pancreata of indicated mice.

To test the efficiency of transfection, we electroporated a reporter plasmid that supports cytomegalovirus promoter driven green fluorescent protein (GFP) expression (Fig. 2a). The target region was labelled with endoscopic marker, allowing identification of the electroporated pancreatic area even after weeks (Fig. 2b). Two days post electroporation (PE), the mice were killed and pancreatic tissue was removed to count GFP-expressing cells. Because of the pronounced fluorescence background in the pancreas, which often makes it difficult to distinguish low-level GFP expression from background signal by immunofluorescence, we have performed immunohistochemistry (IHC) against GFP (Fig. 2c). IHC allowed densitometric and volumetric analyses of multiple step cuts through the whole pancreatic tissue. Two days PE, we counted an average of 750 GFP-positive cells per pancreas (Fig. 2d). GFP-expressing cells were restricted to the electroporated area and could not be found in the other parts of the pancreas.

Histologically, we observed a mild-to-moderate intra- and interlobular infiltration of macrophages and neutrophils and occasional acinar cell vacuolization at the electroporation site at day 2 PE (Fig. 2e). Seven days PE, this focal inter- and intralobular infiltrates contained lymphocytes and macrophages with brownish pigment (presumably phagocytosed endoscopic marker and cell debris) and occasional focal acinar to ductal metaplasia was observed (Fig. 2e). Twenty-one days PE, complete regeneration of the acinar compartment occurred. Inflammatory reactions fully or almost entirely disappeared, with the exception of residual accumulations of macrophages removing endoscopic marker. Caspase-3 staining showed a marked but locally limited increase of acinar cell apoptosis at the site of electroporation 2 days PE, whereas at day 7 PE, apoptosis rates were similar in electroporated and non-electroporated pancreata (Fig. 2f,g). Likewise, mild single-cell necrosis of acinar cells, which was observed 2 days PE, was cleared by day 7 PE. All together, these results show that the electroporation protocol causes only mild tissue damage. Nevertheless, this could, in principle, result in the loss of successfully electroporated cells. The number of ‘long-term survivors' among the successfully transfected cells can, however, not be determined in the experiments described so far because the GFP-expressing plasmids are lost over time.

To address this issue, we next electroporated a phosphoglycerate kinase 1 (PGK)-Cre expression vector into pancreata of *Rosa26*^*mT/mG*^ knock-in mice (Fig. 2h). This allele supports whole-body red fluorescence from a membrane-targeted tdTomato (mT) cassette, which is flanked by loxP sites^30^. Cre-mediated excision of the mT cassette permits expression of a membrane-targeted EGFP (mG) cassette located just downstream. This double-fluorescent system allows direct visualization of both recombined and non-recombined cells at single cell resolution. We used this allele as a reporter to determine the number of successfully transfected long-term surviving pancreatic cells. To this end, we collected pancreata from mice electroporated with PGK-Cre or control vectors 7 days PE and performed endogenous fluorescence-based quantification of cells converted from mT to mG (Fig. 2i,j). Although GFP-positive cells could not be observed upon administration of control vectors (*n*=3 mice), we found that an average of 120 cells per pancreas exhibited a mT to mG switch in mice receiving PGK-Cre vector. To examine Cre-mediated recombination at the genetic level, we performed PCR-based amplification and sequencing of the recombined mT/mG allele (Supplementary Fig. 1). As expected, Cre electroporation resulted in recombination of the mT/mG allele, which was, however, only detectable by nested PCR, reflecting its low frequency. No mT/mG conversion was detectable in pancreata electroporated with a control plasmid, confirming the lack of spontaneous recombination.

Because excessive amounts of Cre protein can be toxic^31^ (which might be the case in the unlikely scenario of extensive Cre plasmid delivered to a cell), it cannot be fully excluded that the number of successfully transfected cells is slightly underestimated in this experiment. However, by combining the data from the transient GFP delivery approach and the mT/mG long-term conversion experiments, we can unequivocally conclude that only a very small fraction of pancreatic cells (maximum a few hundred cells per organ) is targeted and survives in the long-term. We therefore suggest that these electroporation settings for CRISPR/Cas9 delivery might be optimal to induce low-frequency mosaic targeting of somatic cells, a fundamental aspect of sporadic human tumorigenesis.

### Pancreatic cancer induction by multiplexed CRISPR/Cas9

**Figure 3:**
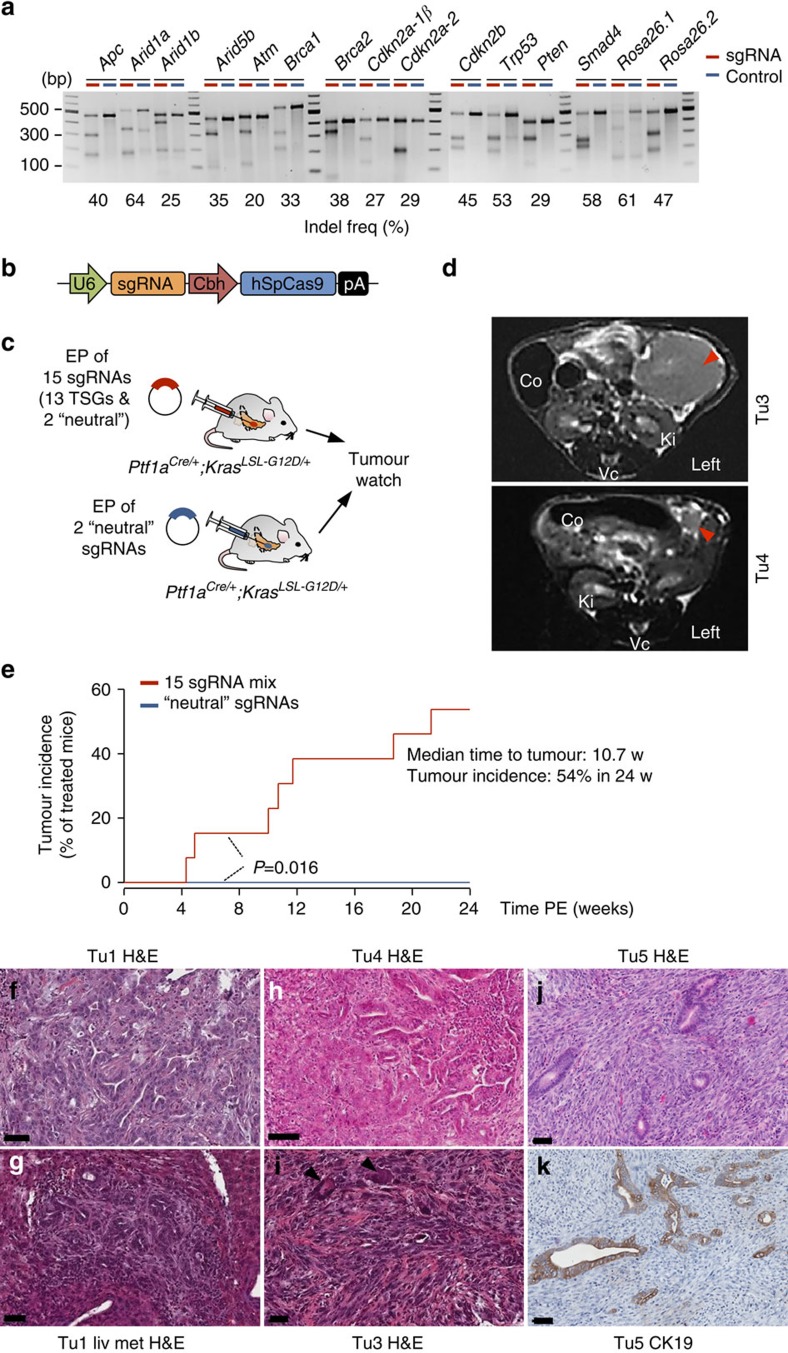
CRISPR/Cas9-based pancreatic multiplex-mutagenesis and cancer induction in *Ptf1a*^*Cre/+*^*;Kras*^*LSL-G12D/+*^ mice. (**a**) *In vitro* surveyor nuclease assays showing cutting efficiencies of sgRNAs chosen for *in vivo* experimentation. Controls were transfected with neutral sgRNAs targeting the *Rosa26* locus. (**b**) A modified version of the pX330 vector was used for transient Cas9 and sgRNA expression. Expression of codon-optimized *S. pyrogenes* Cas9 (hSpCas9) and sgRNAs are driven by the Cbh and the human U6 promoter, respectively. (**c**) Scheme of experimental procedures to generate experimental (top) and control (bottom) cohorts. EP, electroporation. TSG, tumour suppressor gene. (**d**) Tumour formation was monitored regularly using MRI for up to 6 months PE. T2-weighted MRI sections of a large PDAC 21 weeks (w) PE (upper image) and a small PDAC 5 weeks PE. (lower image). Arrow heads indicate PDACs. Co, colon; Ki, kidney; Vc, vertebrate column. (**e**) Tumour incidence of experimental cohorts electroporated with a mixture of 15 sgRNAs (*n*=13) and of control animals electroporated with two neutral sgRNAs (*n*=8). *P*=0.016; log-rank test. (**f**–**k**) Examples of moderately differentiated PDACs (G2–G3; **f**–**h**) and a corresponding liver metastasis (liv met; **g**), as well as poorly differentiated sarcomatoid (G4) PDACs (**i**–**k**) with anaplastic giant cells (arrow heads) in **i**. IHC in Tu5 confirmed the origin of dedifferentiated sarcomatoid cells from CK19-positive ductal structures (**k**). Haematoxylin and eosin (H&E) stained sections are shown in **f**–**k**. Scale bars, 50 μm.

Another major motivation for developing an electroporation-based approach for CRISPR/Cas9 vector delivery was our goal to achieve CRISPR/Cas9 multiplexing. We anticipated that electrophoretic transfection could allow simultaneous delivery of multiple sgRNAs to individual cells. This is difficult or (for high-level multiplexing) even impossible to be achieved by the use of viral delivery approaches. We have chosen to target two neutral genetic loci as well as a set of 13 tumour-suppressor genes, which were previously reported to be involved in pancreatic tumorigenesis, albeit at very different frequencies (Fig. 3a and Supplementary Table 1). sgRNAs were cloned into a modified pX330 (refs 15, 29) vector, which supports expression of Cas9 and sgRNAs by the chicken beta actin (Cbh) and U6 promoters, respectively (Fig. 3b). We tested multiple sgRNAs per locus by transiently transfecting a mouse pancreatic cancer cell line with respective vectors. sgRNAs associated with uniform high cleavage efficiencies were selected for further *in vivo* experimentation. Fig. 3a shows Surveyor assays of these selected sgRNAs.

Over 90% of human pancreatic cancers have *KRAS* mutations and pancreas-specific *Kras*^*G12D*^ expression in *Ptf1a*^*Cre*/+^ (ref. 32); *Kras*^*LSL-G12D/+*^ (ref. 33; PK) mice induces PDAC, albeit after long time periods. We observed a median survival of 472 days (range of 263 to 844 days; *n*=55). To explore the feasibility of CRISPR/Cas9-based tumour-suppressor gene targeting in this model, we performed direct DNA injection and electroporation to co-deliver 15 CRISPR/Cas9 vectors expressing the different sgRNAs. Control mice were electroporated with two ‘neutral' sgRNAs targeting intronic positions of the *Rosa26* locus (Fig. 3c and Supplementary Table 2). All the mice were monitored for tumour development by magnetic resonance imaging (MRI; Fig. 3d). We observed a dramatic acceleration of tumorigenesis in PK mice receiving the 15-sgRNA mix, with animals starting to succumb to pancreatic cancers 4 weeks PE. Seven out of 13 mice (54%) developed tumours within 24 weeks PE in this group, whereas no tumours were detected in PK mice transfected with neutral sgRNAs by MRI (*n*=8; *P*<0.016; log-rank test; Fig. 3e). Examples of pancreatic cancers with different histopathologic characteristics (well/moderately differentiated to undifferentiated or sarcomatoid pancreatic cancers) and liver metastases are shown in Fig. 3f–k. To exclude the possibility that sarcomatoid tumours are in fact sarcomas arising from CRISPR/Cas9 targeted fibroblasts, we performed IHC staining of the epithelial marker cytokeratin 19 (CK19) and E-cadherin. These experiments confirmed the origin of dedifferentiated sarcomatoid cells from CK19-positive pancreatic ductal structures (Fig. 3k). In addition, recombination of the ‘stop' cassette at the *Kras*^*LSL-G12D/+*^ allele (mediated by pancreas-specific *Ptf1a*^*Cre/+*^) was detectable in all primary cell cultures derived from these tumours.

### Multiplexed CRISPR/Cas9 for combinatorial gene targeting

**Figure 4:**
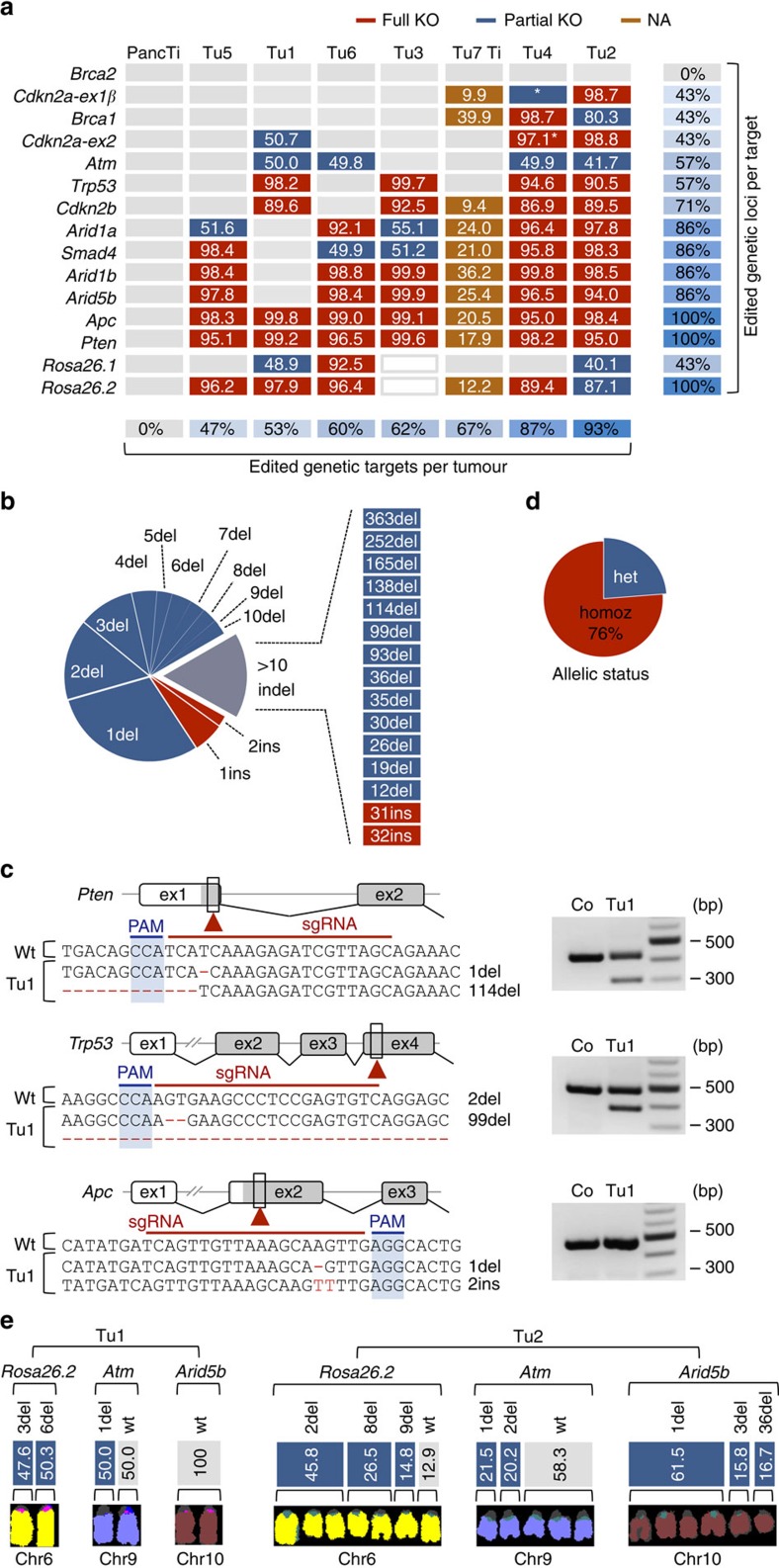
Target site mutations in CRISPR/Cas-induced cancers. (**a**) Indels and allelic status at each target site in electroporated healthy pancreatic tissue (PancTi), PDAC cell lines (Tu1-6) and primary tumour tissue (Tu7 Ti). Numbers in boxes indicate for each target site mutant read frequencies (MRFs; defined as the fraction of mutant sequence reads/all reads at individual target loci). Multiple mutations per target site are presented as one combined MRF. A more detailed presentation of the different mutations at individual target sites is shown in Supplementary Fig. 2. Red and blue boxes indicate complete or partial inactivation of targeted loci, respectively. A target locus was defined to be only partially inactivated if at least one chromosome with non-mutated wild-type sequence was retained. For tissue (brown boxes), assumptions about full/partial inactivation cannot be made. Grey boxes designate a lack of mutations at target sites. The asterisk stands for a large deletion at the *Cdkn2a* locus with fusion of *Cdkn2a-ex1β* and *Cdkn2a-ex2* (see also Fig. 6). White boxes (Tu3) indicate electroporation without *Rosa26*.1 and *Rosa26*.2 control guides. (**b**) Spectrum and distribution of indel types and sizes in all sequenced tumours. Del, deletion; Ins, insertion. (**c**) Examples and sequence context of CRISPR/Cas9-induced homozygous (homoz) mutations at target sites. Large deletions were also detectable by PCR, showing additional shortened products. PAM, protospacer adjacent motive; ex, exon and Co, control. (**d**) Allelic status at target sites across tumours. Homozygousity was defined by a lack of wild-type sequence reads in cancer cell cultures. Het, heterozygous. (**e**) Examples of mutational spectra in a diploid (Tu1) and a poly-/aneuploidy cancer (Tu2). M-FISH and target site sequencing were performed on the cell lines. Results are shown for three representative target genes. MRFs of target site mutations are assigned to individual chromosomes. The existence of more than two mutations at a target site in Tu2 reflects early polyploidization during transient CRISPR/Cas9 expression. Comprehensive data for all chromosomes are shown in Supplementary Fig. 4.

To examine the induction of mutations by CRISPR/Cas9, we performed NGS of PCR-amplified target sites in primary pancreatic tumours and healthy control tissue. Because sequencing reads with large deletions show only poor overlap with the reference genome built, they are often filtered out during mapping by standard bioinformatics tools. We have therefore used manually inspected/mapped capillary sequencing data to develop optimized algorithms for NGS-based high-throughput CRISPR/Cas9-induced indel detection in PCR products (see ‘Methods' section). We have not found mutations (above sequencing error rates) at sgRNA target sites in non-tumour pancreatic tissue surrounding pancreatic cancers in electroporated mice (Fig. 4a). This was expected because of the low number of cells to which CRISPR/Cas9 is delivered by electroporation. Mutations induced in these few cells are not detectable by ultra-deep sequencing, because their frequency is far below the sequencing error rate. In contrast to normal pancreatic tissue, all tumours had high-frequency indels at multiple sgRNA target sites (Fig. 4a and Supplementary Data 1), reflecting clonal expansion of CRISPR/Cas9-induced driver mutations to give rise to cancer. We used NGS to determine mutant read frequencies at target sites (MRFs; defined as the fraction of mutant sequence reads/all reads at individual target loci). In Fig. 4a, multiple mutations at individual target sites are presented in a simplified way as cumulative MRFs in each tumour. A detailed presentation of the type and frequency of mutations at individual target sites is shown in Supplementary Fig. 2 for each cancer.

The size of deletions and insertions ranged from 1 to 363 and 1 to 32 bp, respectively (Fig. 4b). Indel size and frequency correlated inversely. The majority of indels were small and located at the position of the Cas9-induced double-strand break (1–5 bp upstream of PAM). Examples are shown in the relevant sequence context in Fig. 4c. Large indels are also detectable by gel electrophoresis (Fig. 4c). There was a strong bias towards deletions (90%) versus insertions (10%) as a result of repair by non-homologous end joining (Fig. 4b).

Each cancer displayed indels in several target genes, reflecting transfection of the cell of origin with multiple sgRNAs (Fig. 4a). Seven to 14 out of 15 targeted genes, were mutated simultaneously in individual cancers, demonstrating (i) the high efficiency of multiplexed CRISPR/Cas9 vector co-delivery to individual cells and (ii) the high efficiency of gene editing by transiently expressed CRISPR/Cas9 in pancreatic cells. This suggests broad applicability of the method for combinatorial gene targeting in the pancreas, for example, to explore synergistic interactions of cancer genes.

We have recently exploited CRISPR/Cas9 multiplexing for forward genetic screening in the mouse liver^29^. To this end, we used hydrodynamic tail vein injection, which allows multiplexed vector delivery to millions of cells, each obtaining a random combination of few or many sgRNAs. This generates a huge population of cells with enormous genetic complexity, thus supporting forward genetic screening by positive selection.

Electroporation-based multiplexed CRISPR/Cas9 mutagenesis in the pancreas differs however from the method deployed in the liver in two key aspects: First, the number of targeted cells is extremely low (few hundred per pancreas) and second, the efficiency of multiplexed vector delivery to individual cells is extremely high (7–14 sgRNAs per cell; including ‘neutral' sgRNAs; see the high frequency of control *Rosa26* indel induction). Therefore, the total complexity of mutagenesis in this setting is low and not appropriate for large-scale forward genetics (and accordingly, no genotype/phenotype correlations could be made in this small cohort). Instead, the methodology will dramatically change our ability to perform complex hypothesis-driven reverse genetics that is not feasible with other model systems. Studying complex tumour-suppressor gene interactions in a scenario of sporadic tumorigenesis is becoming feasible not only in a high-throughput manner, but without any germline genetic engineering and years of intercrosses. In addition, somatic CRISPR/Cas9 genome engineering in inbred adult mice overcomes the problem of unpredictable phenotypic variation in mixed backgrounds, which is an important confounder in multiallelic crosses of transgenic mice.

### Multiplex-CRISPR/Cas9 for *in vivo* negative-selection screening

The only target gene that was not mutated in CRISPR/Cas9-induced cancers was *Brca2*, despite the fact the *Brca2* sgRNAs are functional and were shown to mediate indel formation with similar efficiencies to other sgRNAs *in vitro* (Fig. 3a). This suggests negative selection of *Brca2* inactivation. Indeed, homozygous *Brca2* inactivation was shown to inhibit Kras^G12D^-dependent PDAC formation, but promoted tumorigenesis in a *Trp53* mutant background, when Trp53-dependent cell cycle checkpoints are altered in the mouse^34^,^35^. In addition, in human pancreatic cancers, somatic *Brca2* inactivation is invariably associated with *P53* mutations^2^. We suspect that similar assumptions might also be true for *Brca1*, for which homozygous disruption was only observed in one CRISPR/Cas9-induced (*Trp53*-mutant) cancer. These data show that for the first time negative-selection screening becomes feasible in the mouse pancreas, providing unique opportunities to address a wide variety of biological questions. The approach could be exploited, for example, to systematically explore synthetic lethality and therapeutic vulnerabilities *in vivo*.

### Allelic status of CRISPR/Cas9-induced mutations

Deducing homo- or heterozygousity of CRISPR/Cas9-induced mutations from sequencing data (MRFs) is complex and can be confounded by different factors. First, the high stromal component of pancreatic cancers and the inter-tumour variability of stromal reactions makes it difficult to assign wild-type sequence reads to cancer versus stromal cells. To address this issue, we have generated primary cell cultures (hereafter referred to as ‘cell lines') from six tumours and compared their MRFs. Supplementary Fig. 3 shows that cumulative MRF values at individual target sites clearly discriminated between full (not wild-type sequence) or heterozygous/partial target gene inactivation in cell lines, but not in cancer tissue.

A second level of complexity arises from the frequent occurrence of poly- and aneuploidy in pancreatic cancer. We have, therefore, also performed multispectral karyotyping (multicolour fluorescence *in situ* hybridization (M-FISH)) of all the cell lines and combined M-FISH/sequencing data to obtain an exact picture of chromosome numbers and mutations related to each target site. Tu1 is an example of a cancer with a stable diploid karyotype. Accordingly, it showed a maximum of two independent indels per target site. Examples of chromosomes/target sites with homo-, hetero- or no mutation are shown in Fig. 4e (the full karyotype and mutation spectrum is shown in Supplementary Fig. 4). In contrast, Tu2 had often more than two indels per target site (up to five; Fig. 4e and Supplementary Fig. 4), which could be reconciled by its poly/aneuploidy karyotype. For example, the frequency of the wild-type and the three different indel sequences at *Rosa26.2* could be assigned to the seven copies of chromosome 6 (Fig. 4e; see Supplementary Fig. 4 for more examples). The indel pattern in this tumour also shows that polyploidy was already present at the time of transient CRISPR/Cas9 expression. Overall, in depth analysis of MRFs in cancer cell cultures revealed that in 76% of cases CRISPR/Cas9 mutagenesis induced complete gene inactivation, whereas in 24%, at least one wild-type allele was retained (Fig. 4d).

### Phylogenetic tracking of metastatic disease

**Figure 5:**
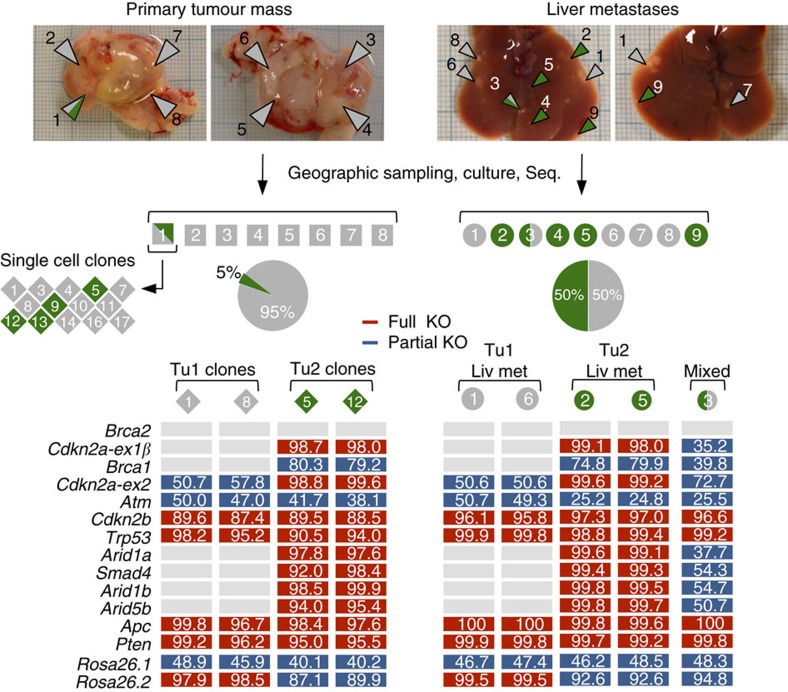
Phylogenetic tracking of CRISPR/Cas9-induced metastatic PDAC. A large (∼1.5 cm) CRISPR/Cas9-induced PDAC was geographically sampled to generate cancer cell cultures (squares) from eight tumour regions. Sequencing (Seq.) of these eight cell cultures suggested the existence of two independent tumours: Tu1, in all cultures (1–8), and Tu2 in culture 1. Further sequencing of 14 single cell clones derived from region 1 (rhombuses) confirmed the existence of two independent primary cancers: see combined MRFs for single cell clones 1 and 8 (Tu1) and 5 and 12 (Tu2). Nine liver metastases (liv met) were collected to generate cancer cell cultures (circles). Mutation patterns showed that both tumours produced metastases to a similar extent. Details about mutation types at target sites are shown in Supplementary Fig. 5.

One limitation of standard pancreatic cancer models (for example, the widely used *Kras*/*Trp53* double-mutant model) is multifocal cancer development, which confounds/limits evolutionary studies or phylogenetic tracking of metastatic spread. We hypothesized that the CRISPR/Cas9 mutation pattern could be exploited to track the evolution of metastases in mice with multiple independent cancers. To this end, we performed extensive geographic sampling of a large tumour mass (∼1.5 cm) in one animal and generated cell cultures from eight tumour regions (Fig. 5 and Supplementary Fig. 5). NGS of target sites in primary cancer tissue and in corresponding cell cultures revealed two independent primary cancers, as defined by (i) the different combination of affected genes and (ii) differences in the indel types and locations at individual target sites. We found that the largest part (95%) of the tumour mass originated from Tu1 (Fig. 5 and Supplementary Fig. 5). Only one out of the eight biopsies (biopsy 1) showed an indel pattern that indicated the presence of an additional tumour. We generated a total of 14 single-cell clones from the primary cell culture of biopsy 1, which made it possible to clearly define Tu2 at the genetic level (Fig. 5 and Supplementary Fig. 5).

We also collected nine metastatic nodules and generated corresponding cell cultures. CRISPR/Cas9-induced indel patterns revealed that both cancers contributed to the metastatic phenotype to a similar extent (50% of nodules originating from Tu1 and 50% from Tu2; Fig. 5 and Supplementary Fig. 5). All together, these results show that CRISPR/Cas9 multiplexing and indel analysis is a very accurate and inexpensive approach (as compared with CGH or exome-sequencing) to phylogenetically track metastases.

### Off-target effects in CRISPR/Cas9-induced cancers

To examine whether transient CRISPR/Cas9 expression leads to off-target cutting, we have sequenced each sgRNA's top five off-target sites and at least three exonic off-targets (Supplementary Tables 3 and 4). The analysis of amplicon-based NGS data from 648 positions (108 potential off-targets per cancer; in six cell lines) revealed exclusively wild-type sequences at these predicted off-target sites. Thus, we conclude that undesired off-target effects are negligible in our experimental setting of transient CRISPR/Cas9 expression.

### Engineering of chromosomal rearrangements by CRISPR/Cas9

Human pancreatic cancer is characterized by a high complexity of chromosomal alterations, including numerical chromosomal aberrations, intra-chromosomal deletions, unbalanced inter-chromosomal translocations or other more complex rearrangements, such as chromothripsis^2^,^6^,^36^,^37^,^38^. The extent of structural variation reported in different studies varies to some extent (possibly because of the use of different pancreatic cancers and methodologies), although all report a high frequency of complex karyotypes. In accordance, we found a median of 139 intra-chromosomal deletions/amplifications and eight inter-chromosomal translocations per tumour in 23 human PDAC cell lines, which we have screened using high-density array comparative genomic hybridisation (aCGH) and M-FISH (Supplementary Fig. 6). Translocations most frequently affected chromosomes harbouring key pancreatic tumour suppressors, such as *CDKN2A* (Chr9) or *P53* (Chr17). In accordance, recent evidence suggests that structural variations in PDAC are non-random: inter-chromosomal translocations, for example, are predominantly unbalanced and typically affect (disrupt) key tumour suppressor genes, including *CKDN2A*, *SMAD4 or P53* (refs 37, 38, 39).

**Figure 6:**
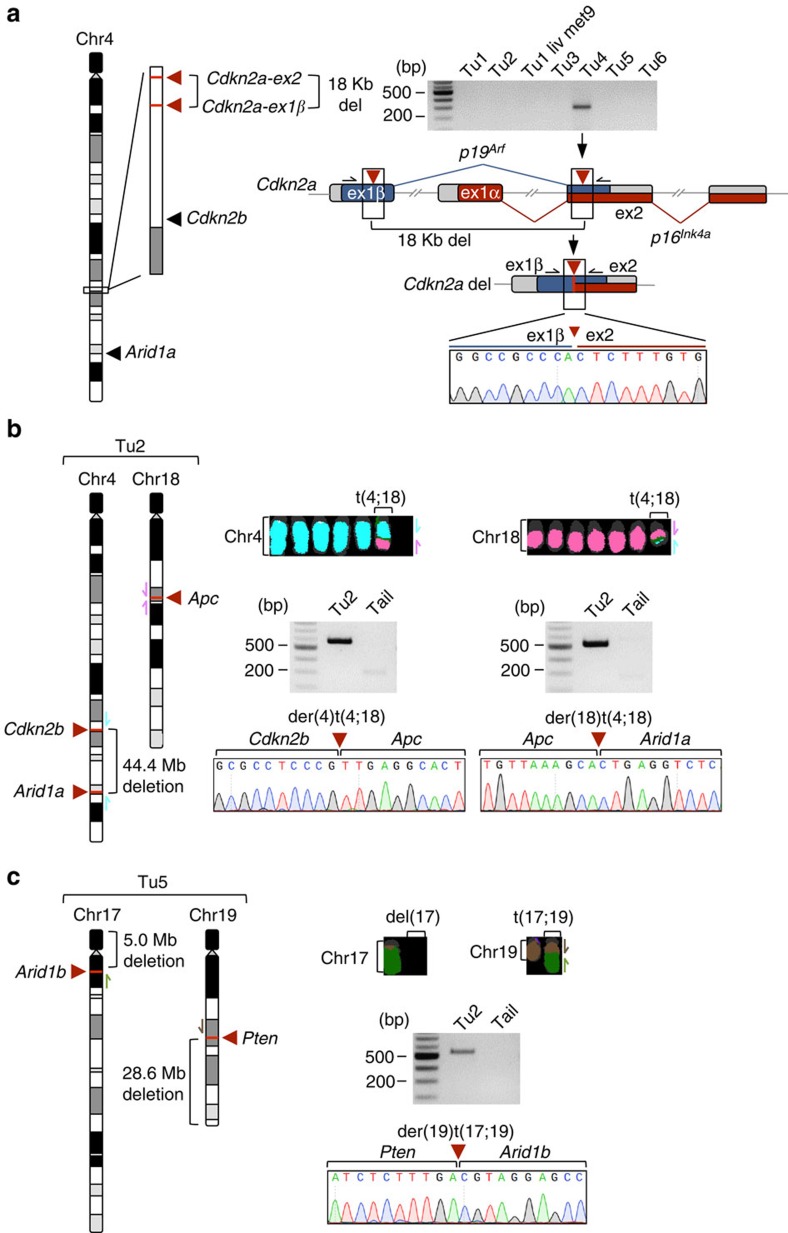
Chromosomal rearrangements in cancers induced by CRISPR/Cas9-multiplexing. (**a**) CRISPR/Cas9-induced intra-chromosomal deletion of an 18 Kb fragment by combinatorial sgRNA targeting. PCR screening for all possible intra-chromosomal fusions on chromosomes with more than one sgRNA target site was performed across pancreatic cancers. The gel image shows respective PCRs at the *Cdkn2a* locus. sgRNA target sites in *Cdkn2a-ex1ß/ex2* and PCR primers are indicated by red arrow heads and black arrows, respectively. Sanger sequencing confirmed the ex1β-ex2 fusion. del, deletion; ex, exon. (**b**,**c**) M-FISH of cell lines revealed unbalanced inter-chromosomal translocations induced by combinatorial CRISPR/Cas9 targeting in two out of six cancers. Fusion products were detected by PCR and sequencing. Arrows indicate primer positions. del, deletion; der, derivative; t, translocation.

We and others have previously shown that CRISPR/Cas9 can induce large intra-chromosomal deletions *in vivo*^29^,^40^,^41^. To comprehensively analyse whether multiplexed CRISPR/Cas9 mutagenesis can induce complex genomic rearrangements in the pancreas, we have first performed a systematic PCR-based screen in all primary tumours for potential intra-chromosomal deletions on chromosomes with more than one sgRNA target site (Supplementary Fig. 7). Out of the nine possible fusions, we found evidence for one 18-Kb deletion in Tu4: caused by sgRNAs targeting *Cdkn2a-ex1β* and *Cdkn2a-ex2*, which lead to the inactivation of both *p16*^*Ink4a*^ and *p19*^*Arf*^ (Fig. 6a).

In addition, we performed aCGH because we have previously observed that the boundaries of large CRISPR/Cas9 deletions occasionally go beyond the sgRNA target sites, and might therefore be missed by the above-described ‘fusion-PCR' screening approach. aCGH also allowed a more comprehensive genome-wide analysis of chromosomal alterations. These experiments confirmed the above-described deletion at the *Cdkn2a* locus in Tu4 and identified another similar deletion in Tu2, which was not detected by the fusion PCR approach (but is very likely to be CRISPR/Cas9 induced). We also found 44.4, 28.6 and 5.0 Mb deletions that were linked to unbalanced translocations, as described below in Tu2 and Tu5. In summary, three out of six tumours had large CRISPR/Cas9-induced deletions (Tu2, Tu4, Tu5), whereas three tumours had ‘silent' aCGH profiles.

To screen for CRISPR/Cas9-induced inter-chromosomal translocations, we performed M-FISH in the six cell lines. We found unbalanced translocations in two out of these six cases. An unbalanced reciprocal translocation in Tu2 involved chromosomes 4 and 18 (Fig. 6b). Sequencing of fusion products revealed a der(4)t(4;18) translocation (fusion between *Cdkn2b* and *Apc*) and a der(18)t(4;18) translocation (fusion between *Apc* and *Arid1a*) (Fig. 6b). A translocation in Tu5 (der(19)t(17;19)) was non-reciprocal (between *Pten* and *Arid1b*; Fig. 6c) and led to loss of parts of Chr17 and Chr19. In all the cases, the translocations were clonal and breakpoints were at the exact sgRNA target sites, confirming that they are CRISPR/Cas9 induced.

On average, there was less than one CRISPR/Cas9-induced deletion or translocation per tumour, which represents only a small fraction of the total number of aberrations observed in *Ptf1a*^*Cre/+*^*;Kras*^*LSL-G12D/+*^*-*induced pancreatic cancers (∼20.5 structural aberrations per tumour; *n*=40; profiling performed by aCGH. In addition, we found that CRISPR/Cas9-dependent chromosomal aberrations did not have a negative impact on the quality of other applications, for example, on the negative-selection screen.

The impact of translocations on genomic instability in pancreatic cancer is unclear. It is striking that the majority of *CDKN2A* and *SMAD4* inactivations in human PDAC are a result of translocations rather than simple intra-chromosomal deletions, as seen in most other cancer types^39^. This raises the possibility that genome imbalance in itself could be an important driver of pancreatic tumorigenesis. Indeed, recent studies in yeast and eukaryotic cells suggested that numerical and unbalanced structural chromosomal aberrations can drive genomic instability (for example, the acquisition of new rearrangements)^42^,^43^. However, functional analysis of such phenomena at an organismal level in the context of cancer has not been possible to date. Although genomic instability can be nonspecifically induced/elevated by genetic changes of stability genes (for example, *Cenpe*, *Mad2*, *Trp53*)^44^, these genes (i) do not allow locus-specific induction of structural variation and (ii) have additional independent cancer-relevant functions, confounding the interpretation of resulting phenotypes.

We show here for the first time that such limitations can be overcome. The structural variations associated with CRISPR/Cas9 tumour suppressor gene inactivation suggests that there might be selective pressure beyond simple gene disruption for complex rearrangements to occur in pancreatic cancer. Our experiments show that multiplexed CRISPR/Cas9 will allow a systematic analysis of such questions directly *in vivo*. In human cancer, the frequency of DNA breakage is the parameter that best predicts the likelihood of a particular genomic site being involved in a translocation^45^. Therefore, we expect that using multiple sgRNAs targeting a specific locus, might substantially increase the efficiency of inducing specific inter-chromosomal translocations.

## Discussion

Our studies show multiplexed somatic genome editing and cancer induction in the pancreas, which will provide access to the genetic complexity of pancreatic cancer. We demonstrate several major types of applications that require multiplexing, including (a) high-throughput functional analysis of complex cancer gene interactions/networks, (b) phylogenetic tracking of metastatic disease, (c) *in vivo* negative selection screening and (d) direct *in vivo* chromosome engineering.

Transfection-based multiplexed CRISPR/Cas9 delivery developed in this study has unique features and a number of advantages over other (for example, viral) delivery approaches^27^,^28^. First, in contrast to viral approaches, it allows highly efficient delivery of multiple sgRNAs per cell to enable the different applications that require multiplexing. Second, the protocol is fast and allows high-throughput studies; there is no need for time-consuming virus production/testing, as naked DNA can be injected. Third, off-target effects are not being observed, presumably because of the transient nature of transfection, which contrasts the long-term Cas9 and sgRNA expression from genome-integrated viral or transposon-mobilized DNA. Fourth, there is no risk of virally infected cells being eliminated by adaptive immunity. Fifth, insertional mutagenesis is not an issue, as sometimes observed with viral or transposon-based delivery approaches. Finally, there is no need for biosafety level two experimentation as required for many viral delivery approaches.

Next generation sequencing of human cancers and genome-wide transposon-based genetic screening studies have revealed the extensive complexity and heterogeneity of the genetic networks underlying pancreatic tumorigenesis^2^,^3^,^4^,^5^,^6^,^7^. Multiplexed CRISPR/Cas9 mutagenesis in adult mice will now allow to systematically validate such data in a high-throughput manner and to interrogate if and how putative cancer genes collaborate during tumour initiation, progression and metastatic spread *in vivo*. This will dramatically enhance our ability to functionally annotate pancreatic cancer genomes and signalling networks. We have targeted tumour suppressor genes in this study, but in principle, oncogenes can be targeted somatically too: either by conditional somatic gene transfer using avian retroviral vectors^46^ or by delivering re-engineered Cas9 as a programmable transcription factor, which can be used to activate gene expression (for example, nuclease-deficient Cas9 fused to the VP64 transactivation domain^47^,^48^).

We developed for the first time an approach for negative-selection screening in the mouse pancreas. As proof-of-principle, we showed selection against *Brca2* inactivation, confirming previous reports on the inhibition of Kras^G12D^-dependent PDAC formation by *Brca2* inactivation (in a *Trp53* proficient context) in the mouse^34^,^35^. Negative-selection screening provides unique opportunities to address a wide range of biological questions. It could be exploited, for example, to discover essential Ras downstream targets and vulnerabilities in pancreatic cancer, or to systematically explore synthetic lethality *in vivo*.

We found that the complex mutational signature of multiplexed CRISPR/Cas9 genome engineering can be exploited for phylogenetic tracking of metastatic disease. In principle, this approach can also be extended to study the timing of systemic dissemination or to develop early detection tools based on the monitoring of circulating tumour DNA. Such CRISPR/Cas9 applications could substantially facilitate not only studies into the genetic basis of cancer evolution and metastasis but also into translational aspects of the disease.

Chromosome engineering has been the biggest challenge in genetic manipulation of the mouse germ line^49^. We found that complex rearrangements were induced by multiplexed CRISPR/Cas9 in a subset of cancers. We observed not only intra-chromosomal deletions but also provide the first example of targeted somatic engineering of inter-chromosomal translocations in a higher organism. This will have a profound impact on our ability to study if and how pancreatic tumorigenesis is driven by chromosomal imbalance, a hallmark of pancreatic cancer. CRISPR/Cas9-based chromosome engineering will also facilitate the functional analysis of PDAC-associated noncoding regions emerging from GWAS and sequencing studies^2^,^50^,^51^. Finally, these results also pinpoint limitations of CRISPR/Cas9 multiplexing in the pancreas, thus providing guidance for its accurate use: particularly in experimental settings where structural rearrangements are not a desired outcome, the level of *in vivo* multiplexing will have limitations and the occurrence of rearrangements will need to be tested for.

Multiplexed CRISPR/Cas9 genome editing will not only increase the speed/efficiency with which pancreatic cancer can be modelled in mice for biological and preclinical research, but the low-frequency mosaic transfection pattern described here also better recapitulates the sporadic nature of human tumorigenesis than traditional knockouts.

## Methods

### Design of sgRNA sequences

Consensus coding sequences (CCDS) for each target gene were downloaded from ensembl.org and sgRNA cassettes generated using the CRISPR design tool (http://crispr.mit.edu). sgRNA sequences are shown in Supplementary Table 5.

### Cloning of *CRISPR-SB*

For delivery of CRISPR/Cas9 components, we used a modified one-vector system pX330 (Addgene #42230) with Sleeping Beauty inverted terminal repeats flanking sgRNA and Cas9 expression cassette (CRISPR-SB)^29^. The transposon repeats allow stable genomic integration of the CRISPR construct when co-delivered with a transposase, an option not pursued in this study. Annealed sgRNA oligonucleotides (Supplementary Table 5) were cloned into *BbsI*-opened CRISPR-SB vector^52^.

### SURVEYOR assay for individual sgRNA cleavage efficiency

To determine individual sgRNA cleavage efficiency in our CRISPR-SB vector system, we used the mouse pancreatic cancer cell line PPT-4072 that contains intact loci at all target sites. The cell line was cultured in RPMI (Thermo Fisher Scientific Inc.) supplemented with 10% fetal bovine serum (Biochrome) and 1 × Pen Strep (penicillin 50 units ml^−1^, streptomycin 50 μg ml^−1^; Thermo Fisher Scientific Inc.). A total of 50,000 cells were seeded in a 12-well plate the day before transfection. 500 ng of respective CRISPR-SB vector and 200 ng of pLentiX1-Puro (Addgene #17297) vector were co-transfected overnight with 25 μl Lipofectime 2000 reagent (Life Technologies). The cells were selected with 4 μg ml^−1^ puromycin (Thermo Fisher Scientific Inc.) for approximately 72 h, lysed with DirectPCR lysis kit (Qiagen) and amplified by TaKaRa *Ex Taq* polymerase (Clontech) using primer pairs listed in Supplementary Table 6. A total of 250 ng of each PCR product was adjusted in a total of 12 μl 1 × TaKaRa reaction buffer. Heteroduplex formation and SURVEYOR nuclease assay (Transgenomic) were performed according to manufacturer's instructions. Cas9-mediated indel frequency was calculated on the basis of the fraction of enzymatically cleaved DNA, as determined by integrated intensity of gel bands^53^.

### Animal experiments

Mouse lines used in this study include *Ptf1a*^*Cre/+*^ (ref. 32), *Kras*^*LSL-G12D/+*^ mice^33^ and *Rosa26*^*mT/mG*^ mice^30^ (all on a mixed C57BL/6J;129S background). Animals were housed and maintained under specific-pathogen-free conditions according to the institutional guidelines. All animal studies were conducted in compliance with European guidelines for the care and use of laboratory animals and were approved by the Institutional Animal Care and Use Committees (IACUC) of Technische Universität München, Regierung von Oberbayern and the UK Home Office.

### Surgical procedures and *in vivo* electroporation

In principle, *in vivo* electroporation-based transfection is widely applicable in many organs, for example, muscle^54^, liver^55^, lung^56^, brain^26^. For surgery, 8-to-15-week-old mice were anaesthetized with a combination of medetomidine (0.5 mg kg^−1^), midazolam (5.0 mg kg^−1^) and fentanyl (0.05 mg kg^−1^, MMF). The left flank was carefully shaved, the eyes protected with ointment and the abdomen disinfected. When anaesthesia was complete, an ∼1.5-cm left-lateral incision caudal to the spleen was made, and the pancreas located, which was then carefully pulled out of the abdomen to make it accessible for intraparenchymal injection. Plasmid mixture was administered slowly using a 27G cannula at a depth of 3–4 mm. Four μg of each CRISPR-SB vector (60 μg for 15 sgRNA mix or 8 μg for Rosa26 sgRNA control mix in a total volume of 50 μl) was delivered. The cannula was left in this position for at least 30 s to avoid leakage of the bleb. For *in vivo* electroporation, the Nepa21 square-pulse generator connected to forceps-type electrodes equipped with 3 mm^2^ disks (CUY650P3, Nepa Gene Co., Ltd., Ichikawa, Chiba, Japan) was used. For electroporation, the injection site was carefully sandwiched by the forceps-type electrodes. To limit tissue damage, a maximum voltage of 50 V was used. In cases where the Joule heat measurement during poring pulses (Pp) was less than 0.15 Joule, we applied a second series of pulses to increase transfection efficiency. The Nepa21 instrument is capable of generating exact square-pulses facilitating efficient DNA transfer to cells *in vivo*. During electrophoretic transfection, two different types of pulses are applied to the organ (Fig. 1b,c). Owing to the higher voltage, the first series of pulses (Poring pulse, Pp) introduces pores into the plasma membrane of the cells. The second series contains lower amplitudes that are extended (Transfer pulse; Tp), which facilitates the transfer of DNA particles into the cytoplasm. After electroporation, the pancreas and spleen were carefully placed back in their anatomical position and covered with 1 × phosphate-buffered saline (PBS; Life Technologies) to avoid organ adhesion. The peritoneum was closed with interrupted sutures (5-0 Ethilon) and the skin with wound clips. The mice were kept in a 37 °C heating chamber until they woke up.

### Efficiency of *in vivo* electroporation in the murine pancreas

Transfection efficiency was tested by injection of 60 μg GFP expressing plasmid pcDNA6.2 (LifeTech), mixed 1:1 with endoscopic marker (GI Supply) into 8-to-15-week-old wild-type mice (C57BL/6J). Endoscopic marker contains a sterile, non-pyrogenic suspension consisting of highly purified carbon particles to create a permanent mark of labelled tissue. To identify GFP-expressing cells, the mice were killed 2 days PE. For dissection, the area of injection was located by endoscopic marker. Pancreatic tissue was fixed in formalin solution, embedded in paraffin and cut in 2 μm sections. Every other section was sampled and IHC against GFP performed (Supplementary Table 7). Overall, 12 out of 24 specimens were counted for GFP-positive cells.

### Long-term surviving transfected pancreatic cells

To determine the number of successfully transfected long-term surviving pancreatic cells 8-to-15-weeks-old *Rosa26*^*mT/mG*^ mice were electroporated using 30 μg PGK-Cre vector (Addgene #11543) or non-vector controls and pancreata were removed 7 days PE. Cre-mediated recombination of the *Rosa26*^*mT/mG*^ allele results in excision of mT (membrane-targeted tdTomato) and expression of mG (membrane-targeted GFP). Tissues were fixated in 4% paraformaldehyde without methanol for 1 h and subsequently dehydrated in 15 and 30% sucrose dissolved in PBS until tissue sunk. Dehydrated tissues were embedded in Tissue-Tek (Sakura) and snap frozen in liquid nitrogen for cryosectioning. To analyse recombination events in the electroporated area of the pancreas, the marked area of injection was completely sampled: every other 10 μm section was analysed. Therefore, specimens were counterstained with DAPI (Thermo Fisher Scientific Inc.) and recombined GFP-positive cells were counted by fluorescence microscopy.

### Nested PCR to detect recombination of the *Rosa26* ^*mT/mG*^ allele

Nested PCR was performed to verify Cre-mediated genetic recombination of the *Rosa26*^*mT/mG*^ allele in reporter mice. DNA from the electroporated site of pancreata was isolated and >100 ng DNA submitted to the first PCR run using *Taq* Polymerase (VWR International; 40 cycles; *T*_a_=62 °C; Supplementary Table 8). One μl of PCR product, or 1 μl of a 1:10^5^ dilution of the positive control, were taken for the second amplification step using the nested primer set (30 cycles; *T*_a_=62 °C; Supplementary Table 8). Nested PCR products were either size separated on a 1.5% agarose gel or purified (QIAquick PCR Purification Kit, Qiagen) for validation of Cre-mediated *Rosa26*^*mT/mG*^ recombination by Sanger Capillary sequencing.

### MRI screening

MRI was performed on a 3-Tesla clinical MRI Philips Healthcare system (Ingenia 3 T) with human eight-channel wrist coil (SENSE Wrist coil 8 elements) following previously described protocol that was adapted for 3 T scanners. Five to 10 weeks post electroporation, the mice were screened once per month by MRI. For this, longitudinal T2-weighted (T2w) turbo spin-echo imaging (slice thickness=0.7 mm, in-plane resolution=0.3 × 0.38 mm^2^, TR/TE=4,352 ms/101 ms, TF=21, NSA=9) was performed for tumour detection. The mice were killed when tumours reached a size larger than 2 mm diameter^57^.

### Histology and Immunohistochemistry

Preparation of formalin-fixed paraffin-embedded tissue was performed as described above for efficiency testing. Specimens were haematoxylin and eosin stained according to standard protocols. Pretreatment procedures, primary antibodies and dilutions for IHC are listed in Supplementary Table 7. For visualization, either horseradish peroxidase-labelled secondary rabbit anti-rat antibody (1:1,000, Jackson Immuno Research) or secondary mouse anti-rabbit antibody (1:300, Dako) were used and detection was performed by following the manual of the Bond Polymer Refine Detection Kit on a Bond Max staining roboter (Leica).

### Establishment of primary cell lines

For the isolation of primary murine cell lines, tumour tissue from the primary tumour or liver metastases were washed in PBS, cut into small pieces, transferred in collagenase digestion media (200 U ml^−1^ Collagenase Type II (Worthington), 10% FBS, 1 × Pen Strep (penicillin 50 units ml^−1^, streptomycin 50 μg ml^−1^), 1% Fungizone (2.5 μg ml^−1^) in RPMI 1640 medium (all Thermo Fisher Scientific Inc.)) and digested overnight at 37 °C. The following day, released cells were seeded in a six-well plate and passaged in RPMI 1640 medium containing 10% FBS, 1 × Pen Strep and 1% Fungizone.

### DNA isolation and Sanger sequencing

DNA was isolated from tissue samples (stored at −20 °C in RNAlater (Sigma-Aldrich)) or frozen cell pellets of corresponding cell lines using the DNeasy Blood and tissue kit (Qiagen). Amplification of target genomic regions was performed using Q5 High-Fidelity DNA Polymerase (New England Biolabs). For Sanger capillary sequencing, the PCR products were purified (QIAquick PCR Purification Kit, Qiagen) and each PCR reaction was sequenced individually using the corresponding forward primer. Primers were chosen to amplify a PCR product of approximately 400 to 500 base pairs around the target site (Supplementary Table 6).

### Next-generation amplicon sequencing

DNA extraction and amplification of genomic target (Supplementary Table 6) or off-target loci (Supplementary Tables 3 and 4) was performed as described for Sanger sequencing. All amplified target loci (20 μl reaction volume) were pooled and purified (QIAquick PCR Purification Kit, Qiagen). Library preparation with NEBNext Ultra DNA Library Prep Kit for Illumina and quantification was conducted as described previously^58^. Briefly, after end repair and A-tailing, Illumina paired end adaptors were ligated and the individual sample pools were barcoded with 12 cycles of PCR (sequences in Supplementary Table 6). Barcoded samples were pooled and the final library quantified with qPCR for sequencing on Illumina MiSeq (300 bp, paired end).

### Bioinformatic analyses

MiSeq Illumina paired 300 nucleotide reads were mapped with BBMAP short read aligner (http://bbmap.sourceforge.net) using default settings onto mm10 assembly. Only this particular aligner was able to map correctly large deletions >100 bp in comparison to a number of other aligners tested. BAM files were sorted and indexed with samtools (v0.1.19; ref. 59). After mapping, only paired reads (about 3% were unpaired) were extracted based on bitwise flag 0 × 2 resulting in BAM files containing only correctly paired reads. Samtools (v0.1.6) pileup command with option (-i) was used to display lines containing indels to obtain data in pileup format with the number of reads covering sites. Pileup files were processed with VarScan (v2.3.6) pileup2indel command^60^. Detected indels were only considered to be true if supported by more than 100 mutant reads. A list of all filtered indels used for mutation analysis is given in Supplementary Data.

### Intra- and inter-chromosomal rearrangement PCRs

Every possible intra-chromosomal combination of the CRISPR-SB sgRNAs was tested by performing TaKaRa *Ex Taq* PCR on all the tumour samples. Inter-chromosomal rearrangements (translocations) were tested as indicated by M-FISH. In brief, 50 ng of genomic DNA was used for 30 μl PCR reaction volume with primer pairs indicated in Supplementary Table 6. The resulting PCR product was gel purified (Gel purification Kit, Qiagen) and sequenced by Sanger Capillary sequencing.

### M-FISH analysis

To analyse inter-chromosomal fusions/rearrangements in pancreatic tumour cell lines derived from mice electroporated with CRISPR-SB vectors, multicolour fluorescence *in situ* hybridization (M-FISH) was carried out as described before^61^.

## Additional information

**How to cite this article:** Maresch, R. *et al.* Multiplexed pancreatic genome engineering and cancer induction by transfection-based CRISPR/Cas9 delivery in mice. *Nat. Commun.* 7:10770 doi: 10.1038/ncomms10770 (2016).

## Supplementary Material

Supplementary Information Supplementary Figures 1-7, Supplementary Tables 1-8 and Supplementary References.

Supplementary Data 1 Supplementary Data: Indels identified by amplicon sequencing at sgRNA target sites in CRISPR/Cas9-induced pancreatic cancers and cancer cell lines.
