# Supplementary material for: Multiplexed pancreatic genome engineering and cancer induction by transfection-based CRISPR/Cas9 delivery in mice
Source: Nat Commun. 2016 Feb 26;7:10770. doi: 10.1038/ncomms10770 (PMC4773438; doi:10.1038/ncomms10770)
Supplement: Supplementary Information — Supplementary Figures 1-7, Supplementary Tables 1-8 and Supplementary References. [file ncomms10770-s1.pdf]

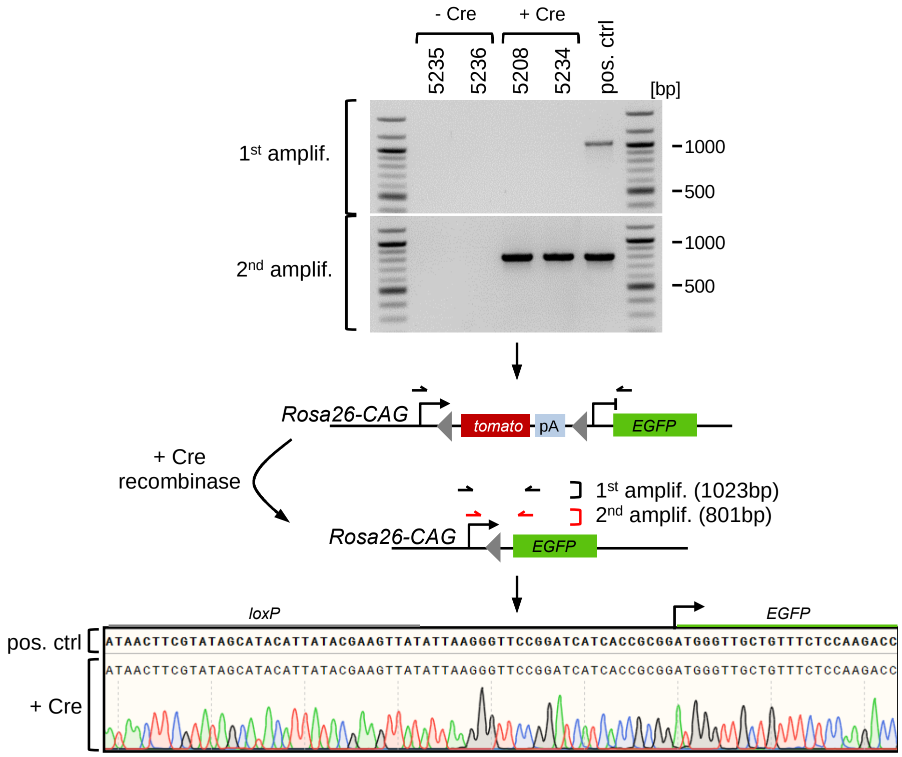

Supplementary Figure 1 | Analysis of *Rosa26*$^{mT/mG}$ recombination in the mouse pancreas upon electroporation-based somatic Cre delivery. Pancreata of four *Rosa26*$^{mT/mG}$ mice were electroporated with or without PGK-Cre plasmid and removed 7 days post electroporation (PE). Nested PCR was used to detect recombination at the *Rosa26*$^{mT/mG}$ allele. Primer locations are indicated by arrows. Fully recombined pancreata from *Ptf1a*$^{Cre/+}$;*Rosa26*$^{mT/mG}$ mice were used as positive controls. Only this positive control gave a recombination band in the 1st PCR step, whereas in the second/nested PCR reaction Cre-electroporated pancreata also produced a band. For the second/nested PCR reaction input DNA (PCR product) of the positive control was diluted 1:10^5 as compared to the other samples (see materials and methods). PCR bands were sequenced to confirm deletion of the stop cassette. All together these results confirm the extremely low number of pancreatic cells to which plasmid DNA is delivered upon electroporation.

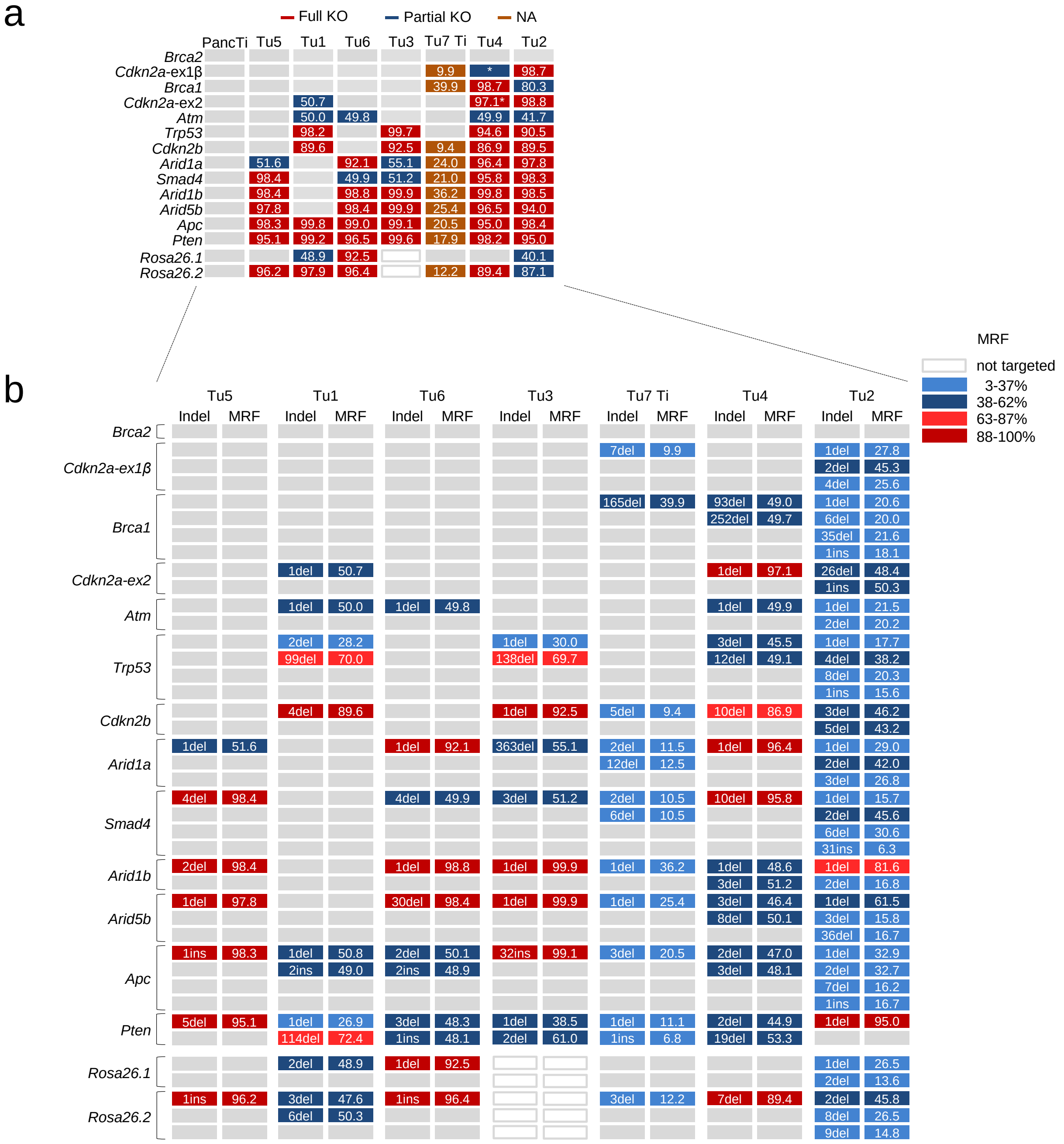

Supplementary Figure 2 | Indels induced by multiplexed CRISPR/Cas9 in primary pancreatic tumours. (**a**) Indels and allelic status at each target site in electroporated healthy pancreatic tissue (PancTi), PDAC cancer cell cultures (Tu1-6) and primary tumour tissue (Tu7; for which cell culture was not successful), as also shown in Figure 4A. Numbers in boxes indicate for each target site mutant read frequencies (MRFs; defined as the fraction of mutant sequence reads/all reads at individual target loci). Multiple mutations per target site are presented as one combined MRF. The asterisk stands for a large deletion at the *Cdkn2a* locus

with fusion of *Cdkn2a-ex1β* and *Cdkn2a-ex2* (see also Figure 6). **(b)** Detailed presentation of individual mutations and their MRFs at target sites for each tumour.

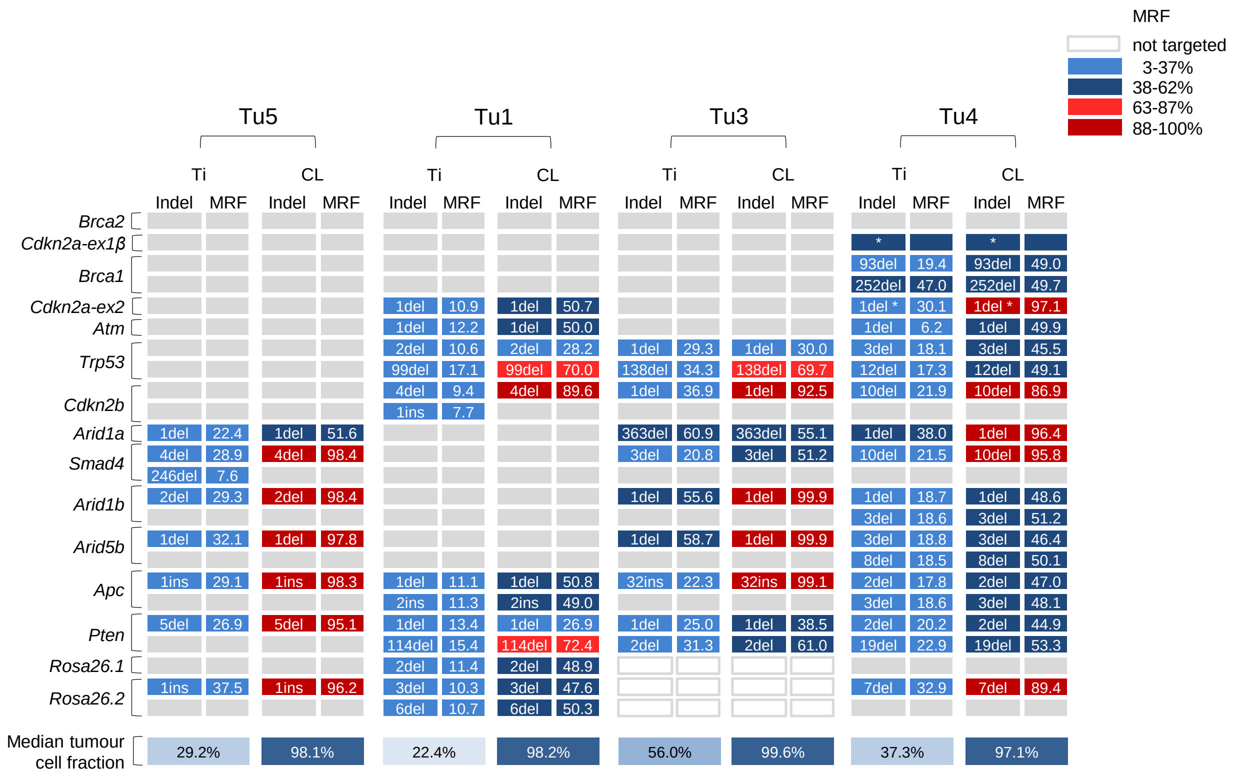

Supplementary Figure 3 | Comparison of CRISPR/Cas9 induced indels in pancreatic tumour tissue and corresponding cell lines. Indels and corresponding MRFs are presented for each target site. The comparison allowed estimation of the median tumour cell fraction in primary pancreatic cancer tissue (only homozygous mutations were used for these calculations). The median tumour cell content for tissue samples ranged between 22 and 56 percent. In contrast, cell cultures presented pure cancer cell populations allowing discrimination of hetero- or homozygous gene inactivation.

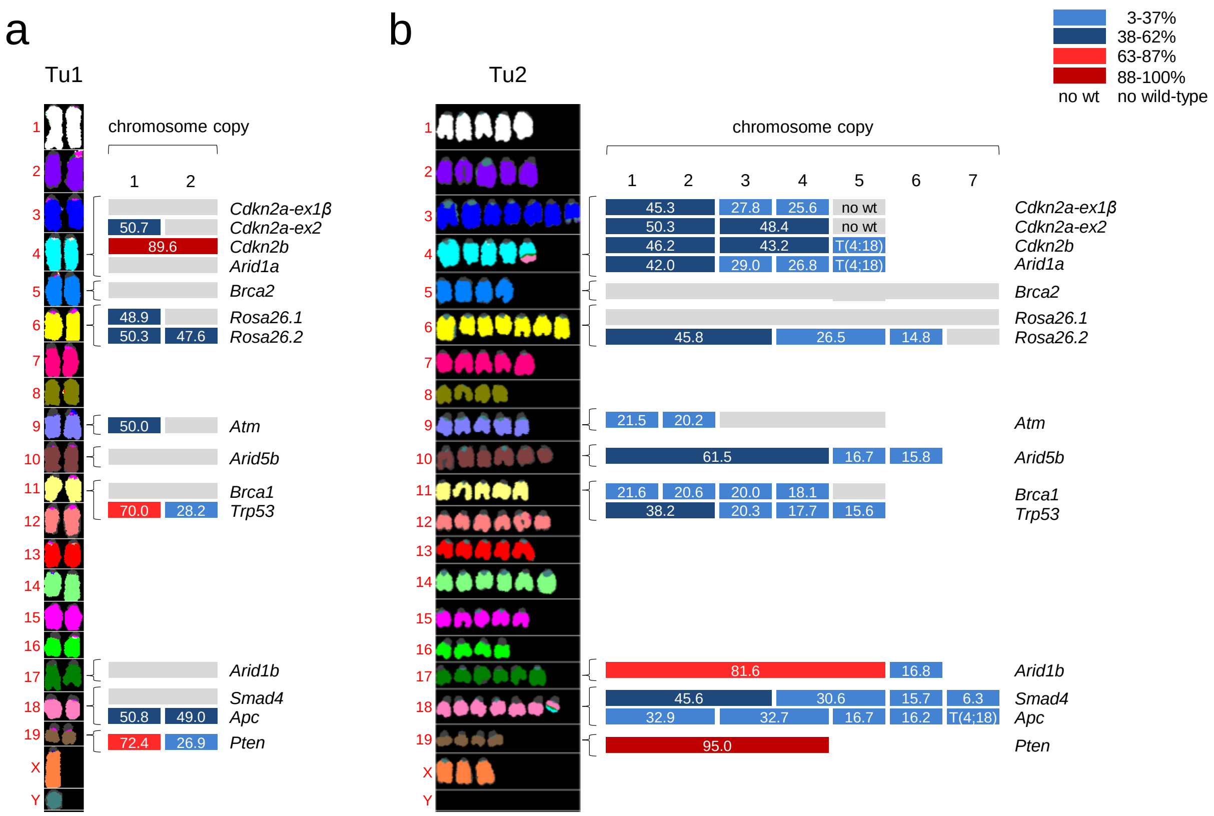

Supplementary Figure 4 | Examples of mutational spectra in a diploid (Tu1) and a poly-/aneuploid cancer (Tu2). M-FISH and next generation sequencing of target sites were performed on primary cancer cell cultures. For each target site distinct mutations and their corresponding MRFs are visualized in individual boxes, which are assigned to individual chromosomes. **(a)** In the scenario of a diploid genome the inactivation type (homo- or heterozygous) can be easily inferred from MRFs. In *Cdkn2a-ex2*, *Rosa26.1* and *Atm*, there was only one type of mutation at target sites and MRFs were ~50%, reflecting heterozygosity. Conversely, homozygous target gene inactivation of *Cdkn2b*, *Rosa26.2*, *Trp53*, *Apc* and *Pten* is a result of one or two distinct mutations at each locus with a total MRF of 100% (lack of wild-type reads). (**b**) In tumours with a complex poly-/aneuploid genome, interpretation of MRFs is more difficult but can be fully explained and reconciled with underlying karyotypes. For example, four distinct *Brca1* mutations (each MRFs of ~20%) were identified in Tu2, which also had ~20% wild-type *Brca1* sequence. M-FISH revealed pentaploidy of Chr11, explaining the MRF pattern. The existence of more than 2 mutations at target sites in Tu2 reflects early polyploidization during transient CRISPR/Cas9 expression.

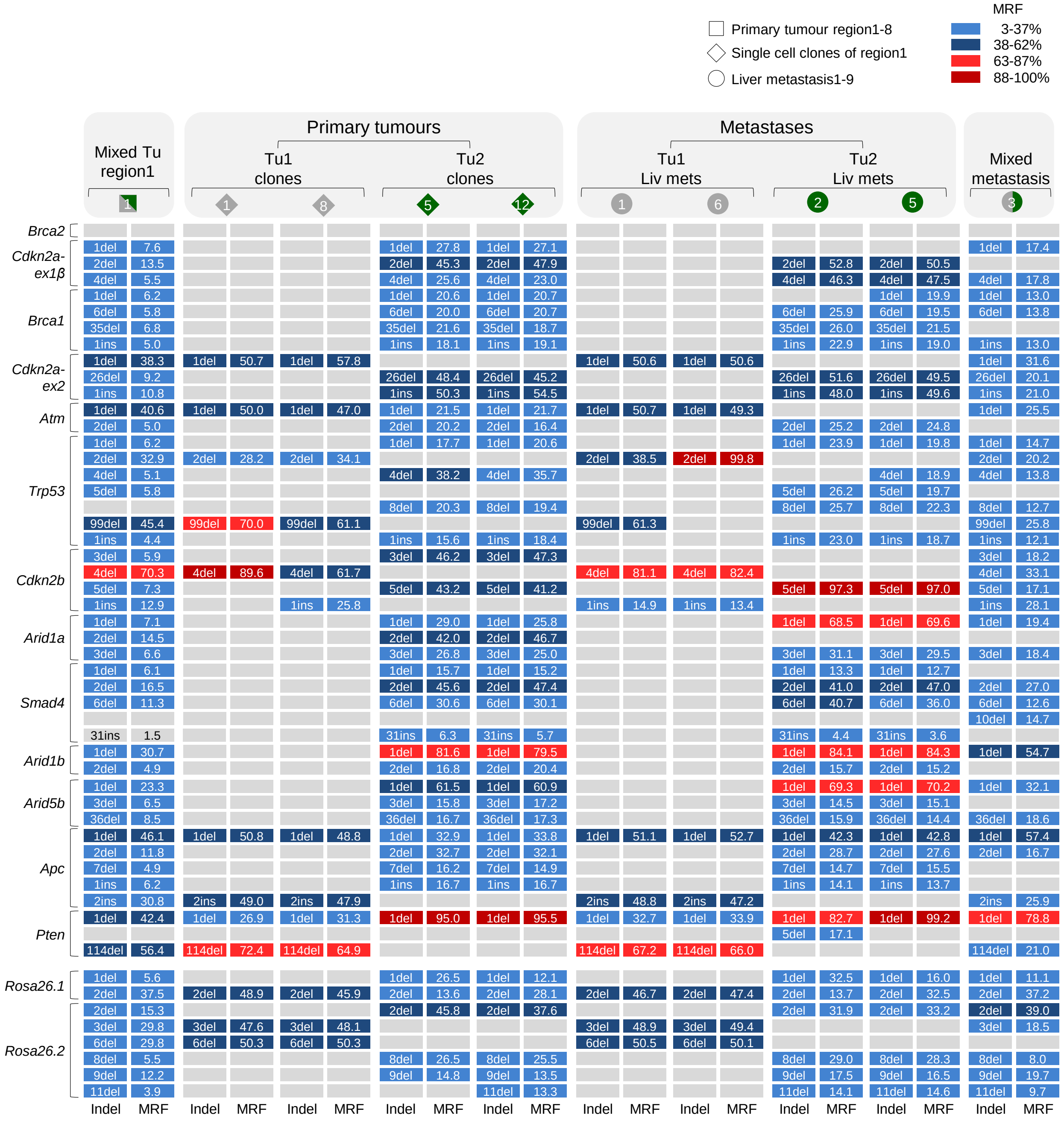

Supplementary Figure 5 | Phylogenetic tracking of CRISPR/Cas9-induced metastatic PDAC. This Supplementary Figure is related to Figure 5, which shows cumulative MRFs at target sites in two independent primary and metastatic PDAC cell lines derived from one mouse. Here, indels and corresponding MRFs are presented in detail for each target site. Note that although different single cell clones derived from individual tumours had largely identical indel patterns, some minor differences could be occasionally observed. For example, most of the Tu1 clones had a 4del and a 1ins mutation at *Cdkn2b* (shown for Tu1-clone8). However, Tu1-clone1 lost the 1ins indel, thereby gaining “homozygosity” for 4del.

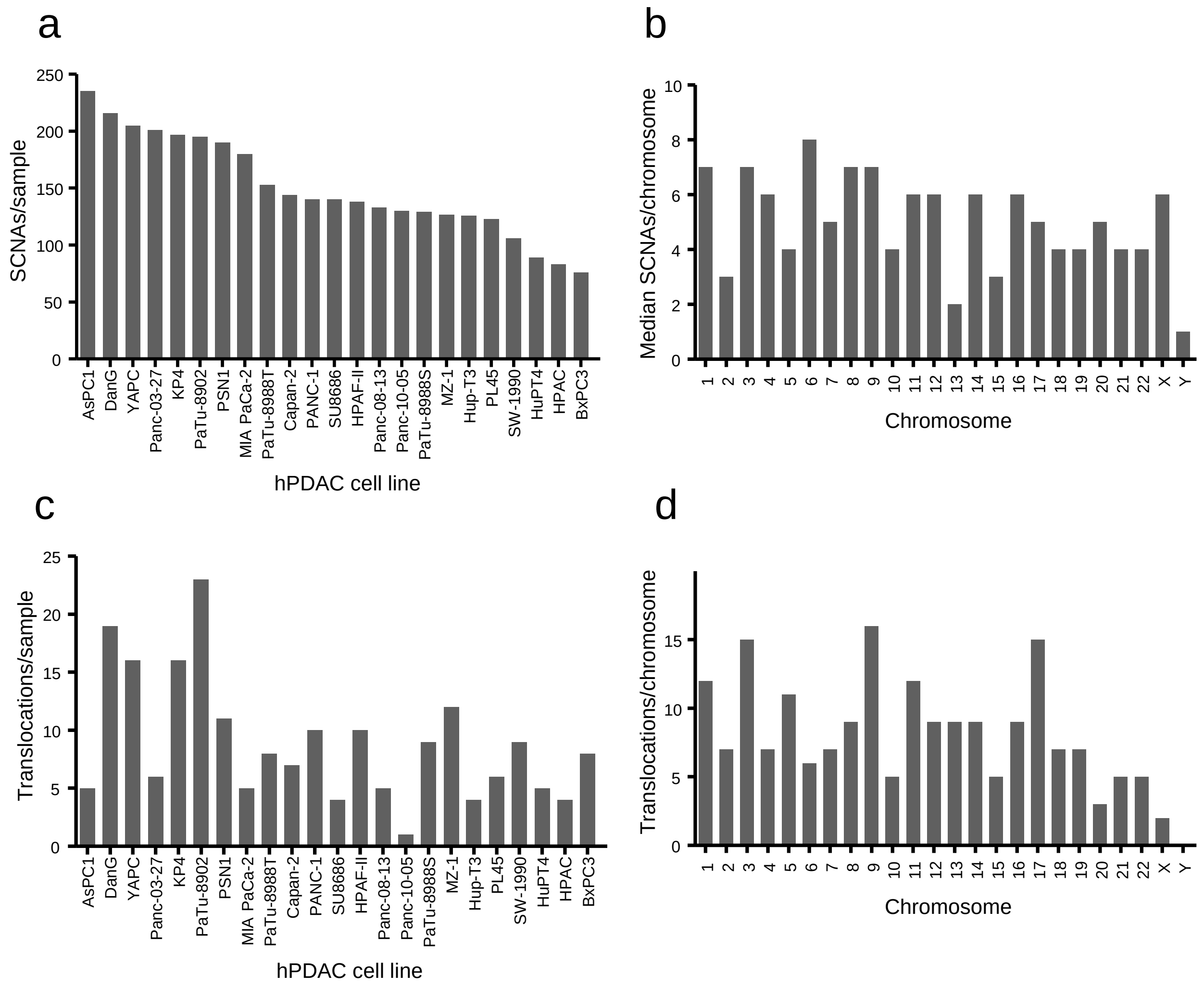

Supplementary Figure 6 | Somatic copy number alterations (SCNAs) and translocations in human PDAC cell lines. A set of 23 human PDAC cell lines has been analysed for SCNAs (deletions or amplifications) and translocations by high-density array CGH and M-FISH, respectively. **(a)** Total number of all SCNAs detected in each cell line. Cell lines are sorted by SCNA frequency. **(b)** Median number of SCNAs observed in all cell lines shown for individual chromosomes. **(c)** Total number of all translocations identified in each cell line. Cell lines are listed as given in (a). **(d)** Total number of translocations observed in all cell lines shown for individual chromosomes.

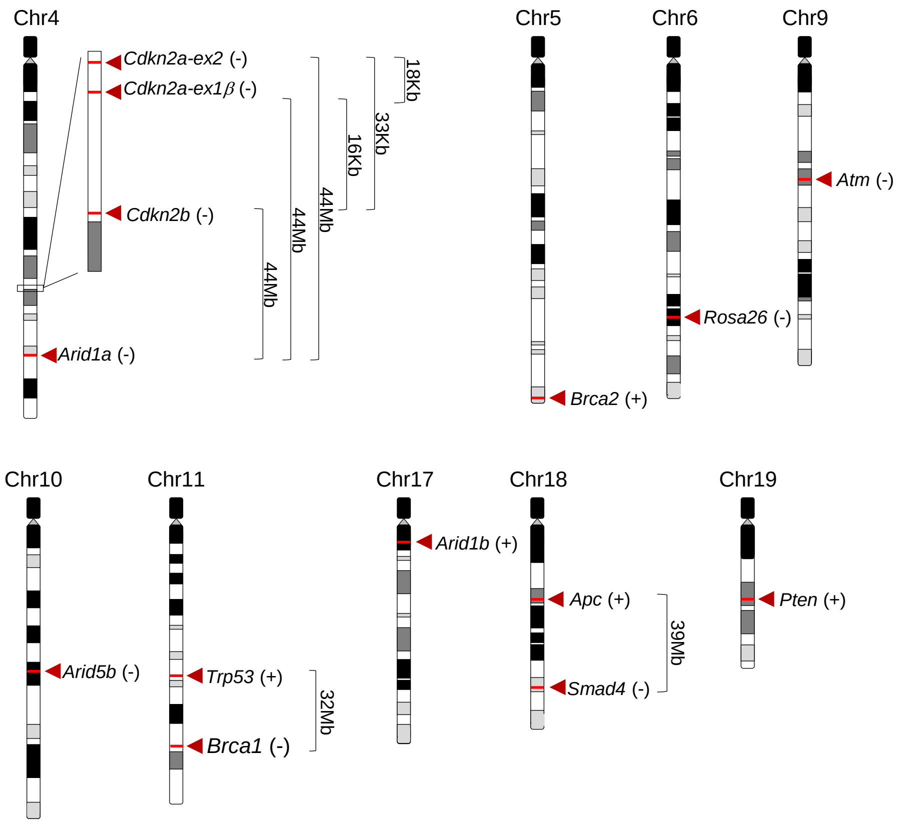

Supplementary Figure 7 | Chromosomal localisation of sgRNA target sites. Scheme of all targeted sites and potential intra-chromosomal deletions resulting from combinatorial target site cleavage in the 15 sgRNA multiplexing experiments performed in this study. Red arrow heads indicate sgRNA target sites and brackets mark the size of potential intra-chromosomal deletions. All pancreatic cancers were screened for fusion events resulting from indicated deletions.

Gene	Type	Range	Reference(s)
APC	MUT	9%	(2/23)# [1]
	DEL, METH, LOSS	48%, 56%, 58%	(12/25, 24/43, 14/24)[2]
ARID1A	MUT	9%	(2/23)# [1]
	MUT	4%	(4/99)[3]
	MUT	8%	(2/24)[4]
ARID1B	DEL	4%	(3/77)[5]
ARID5B	---	---	---
ATM	MUT	4%	(1/23)# [1]
	MUT	5%	(5/99)[3]
BRCA1	MUT	13%	(15/102)* [6]
BRCA2	MUT	10%	(4/41)* [7]
	MUT	10%	(4/39)* [8]
	MUT	4%	(1/23)# [1]
CDKN2A	DEL, MUT	41%, 38%	(15/37, 14/37)[9]
	DEL, MUT, PM	48%, 34%, 14%	(24/50, 17/50,7/50)[10]
CDKN2B	---	---	---
P53	MUT	70%	(19/27)[11]
	MUT	33%	(33/99)[3]
	MUT	75%	(18/24)[4]
	MUT	13%	(3/23)# [1]
PTEN	LOSS	63%	(5/8)[12]
SMAD4	LOSS, MUT, DEL	37%	(31/84)[13]

#	Acinar Cell Carcinoma
*	Germline mutation
LOSS	Loss of expression
DEL	Deleted
MUT	Mutated
PM	Promoter methylation
METH	Gene methylation

Supplementary Table 1 | Tumour suppressor genes previously reported to be involved in pancreatic tumourigenesis. All tumour suppressor genes targeted by CRISPR/Cas9 as well as their inactivation type and frequency of inactivation (range) in human pancreatic cancer are given for each reference. Numbers in brackets correspond to mutated cases per total cases included within each individual study.

Mouse ID	Gender	Tumor ID	First tumour diagnosis (weeks)	Sacrificed (weeks)	Tumour histology
2 "neutral" sgRNA mice					
PPK 203	male	-	No	No	-
PPK 211	male	-	No	No	-
PPK 250	male	-	No	No	-
Pank0032	female	-	No	No	-
Pank0012	female	-	No	No	-
Pank0101	female	-	No	No	-
Pank0103	female	-	No	No	-
ÖR0912	female	-	No	No	-
15 sgRNA mix mice (13 tumour suppressor genes and 2 „neutral“)					
Pank 21	male	Tu4	4.9	5	G2-3 PDAC
Pank 31	male	Tu5	10	10	G4 PDAC/Sarcomatoid
PPK 4f	female	-	No	No	-
PPK 15f	female	-	No	No	-
PPK 40f	female	Tu6, Tu7	11.7	12	G4 PDAC/Sarcomatoid
PPK 103f	female	N/a	4.3	12	G2-3 PDAC
PPK 4m	male	-	No	No	-
PPK 5m	male	-	No	No	-
PPK 6m	male	N/a	18.7	20.6	G2 PDAC
MZ1504	female	-	No	No	-
E2109	male	Tu3	21.3	22	G4 PDAC/Sarcomatoid
MZ1501	male	Tu1, Tu2	N/a	10.7	G2-3 PDAC
MZ1438	male	-	N/a	10	-

Supplementary Table 2 | Acceleration of pancreatic tumour formation in *Ptf1a*$^{Cre/+}$;*Kras*$^{LSL\text{-}G12D/+}$ mice after somatic delivery of multiple sgRNAs by electroporation. Pancreatic cancers were either diagnosed by MRI-based imaging or by macroscopic inspection of isolated tumours. Control mice were electroporated with two guides targeting "neutral" *Rosa26* locus. Additionally to *Rosa26*-targeting control guides, experimental cohort was also injected with 13 sgRNAs binding relevant tumour suppressor genes in PC. (N/a, not analysed).

Name	Chr	Str.	Position (mm10)	Genomic sequence at off-target site	Number of mis-matches	Score	Gene
Apc							
OT_Apc_1	Chr9	-1	26933897	TCAGTTATTAAAGCAAATTGGGG	2	2.27	None
OT_Apc_2	Chr14	1	77604446	GCAGTTGAGAAAGCAAGTTGGAG	3	1.63	None
OT_Apc_3	Chr3	-1	114376816	TCAGATTATAAAGCAAGTTGTGG	3	1.62	None
OT_Apc_4	Chr5	1	75817666	TTAGCTGTTAAAGCAAGTTACAG	3	1.49	None
OT_Apc_5	Chr10	1	94379839	TCAGATGGGAAAGCAAGTTGCAG	3	1.48	None
OT_Apc_6	Chr13	1	106857412	TAAGTTGCTATAGCAACTTGAAG	4	0.34	NM_029665
OT_Apc_7	Chr7	1	12984855	TCAGTTCCTACAGCAAGTTCCAG	4	0.24	NM_001168561
OT_Apc_8	Chr10	-1	52195004	AGAGTTCTTAAAGCAAGGTGGAG	4	0.22	NM_011282
Arid1a							
OT_Arid1a_1	Chr17	1	27452698	CCAGGCCCACCATATGGCTGAGG	4	0.42	None
OT_Arid1a_2	Chr5	-1	122707741	TGAGCCCCACTTTACGGCTGCGG	4	0.40	NM_011026
OT_Arid1a_3	Chr8	1	89565625	TGGGACCCACCATACCGCTGTGG	4	0.26	None
OT_Arid1a_4	Chr16	-1	13032943	TTAAACCCACCATACGCCTAAAG	4	0.26	None
OT_Arid1a_5	Chr8	-1	126296723	ATAGTCCATCCATAGGGCTGAAG	4	0.25	None
OT_Arid1a_6	Chr15	1	79917925	TGTGTCCCACCACAAGGCTGGAG	4	0.16	NM_144811
OT_Arid1a_7	Chr1	1	164211867	ATAGACCCACCCTTCGGCTGGAG	4	0.11	NM_007976
Arid1b							
OT_Arid1b_1	Chr18	-1	34860543	CCGCCCACCTCGGGGACCGTGGG	4	0.79	None
OT_Arid1b_2	Chr1	1	166651815	CTAGGCAACTGGGGGACCCTGAG	4	0.50	None
OT_Arid1b_3	Chr12	-1	21167366	CTGAGCACACTGGGGACCGTGAG	4	0.43	None
OT_Arid1b_4	Chr16	-1	85798156	CTGGGGACCATGGGGACCGTGGG	4	0.43	NM_009621
OT_Arid1b_5	Chr9	-1	45838687	CTTTGCAGCTTGGGGACCGACAG	4	0.37	NM_011792
OT_Arid1b_6	Chr9	1	49250583	CTCAGCTCCTGGGGGACCCTGGG	4	0.34	NM_177701
Arid5b							
OT_Arid5b_1	Chr16	-1	64165874	TCTATGCAAGTCAGATCCTTTAG	3	1.06	None
OT_Arid5b_2	Chr13	-1	8753315	GCTATGCCACTCTGATCCTTCAG	3	0.89	None
OT_Arid5b_3	Chr10	-1	87808962	GCTGTTCAAATCGGATCCTGTAG	3	0.85	None
OT_Arid5b_4	Chr3	1	20285892	AGTTTGCAAATGGGATCCTTGGG	4	0.73	None
OT_Arid5b_5	Chr1	1	107504467	CCTATGCAAATCTGATCCTAAAG	3	0.60	None
OT_Arid5b_6	Chr7	1	123235828	CCTGTGCAAATCCTATCCTTTGG	4	0.09	NM_146198
OT_Arid5b_7	Chr6	1	125679494	CCTATGCAAATTGTATCCCTCAG	4	0.04	NM_011708
Atm							
OT_Atm_1	Chr13	-1	46896751	TGTTATCAACTACCGAAAACTAG	4	0.72	None
OT_Atm_2	Chr10	-1	80700847	ACCTGAGAACTTCCGAAAACCAG	4	0.56	None
OT_Atm_3	Chr13	-1	37858202	CTCTGAAAACTTCCGAAAACTGG	4	0.56	None
OT_Atm_4	Chr3	1	120488144	GATTGTCTACTTCCCAAAACTGG	4	0.40	None
OT_Atm_5	Chr7	-1	49024377	GACTGTCAACTTCCTAAAAACAG	3	0.40	None
OT_Atm_6	Chr9	-1	116131633	GACTGTCCACTTGCGACAACCAG	4	0.24	NM_029575
OT_Atm_7	Chr18	-1	67878014	GGCACTCAACATCCCAAAACTGG	4	0.22	NM_027556
OT_Atm_8	Chr9	-1	59327354	GGCTATCAGCTGCCTAAAACGAG	4	0.12	NM_175325
Brca1							
OT_Brca1_1	Chr18	1	24000244	AAATCTTGGAGTGTCCGGTCAAG	2	1.69	None
OT_Brca1_2	Chr10	-1	125354400	AAATTTTAGTGTGTCCCATCAAG	3	1.05	None
OT_Brca1_3	Chr11	1	111491635	AATTCTTAGAATGTCCCATCCAG	3	0.66	None
OT_Brca1_4	Chr5	-1	34202516	ACATCTGTGAGTGTCCCATCCAG	4	0.42	None
OT_Brca1_5	Chr3	-1	7612625	AAGTCTGGGAGTTTCCGATCCAG	4	0.38	None

OT_Brca1_6	Chr17	1	35127508	GAACCTTGGAGTGTCCGCTCAAG	4	0.32	NM_033477
OT_Brca1_7	Chr12	1	110855953	ACATCTTACAGTGTCAGATGGGG	4	0.07	NM_001199785
OT_Brca1_8	Chr1	1	60486355	AAATCTTAAATTGTCTTATCTGG	4	0.03	NM_001045513
Brca2							
OT_Brca2_1	Chr16	1	7627804	TATGACCAATGAGCCTCAATAAG	3	1.46	None
OT_Brca2_2	Chr5	-1	37348530	GATGACCCATAAGCCTCAAAGAG	4	0.70	NM_145920
OT_Brca2_3	Chr7	-1	83675639	TGGAACAGCTAAGCCTCAATCAG	4	0.58	None
OT_Brca2_4	Chr6	1	101239153	TTAGACCTATAAACCTCAATGAG	4	0.56	None
OT_Brca2_5	ChrX	-1	10836554	GAGAACTGATTAGCCTCAATAGG	4	0.55	None
OT_Brca2_6	Chr12	-1	36476693	AAGGACAGATAAACCTCATTAGG	4	0.14	NM_178629
OT_Brca2_7	Chr5	-1	123819253	TAGGAACGCTACGGCTCAATAGG	4	0.04	NM_001042421
Cdkn2a-ex1ß							
OT_Cdkn2a-ex1ß_1	Chr8	1	44728100	TGATTAAGTTCGTGAGATCCTGG	3	0.79	None
OT_Cdkn2a-ex1ß_2	Chr2	-1	30208726	CAGTGAAGTGCCTGCGATCCCAG	4	0.67	None
OT_Cdkn2a-ex1ß_4	Chr6	-1	87833508	AGGTGTGGTGCGTGCGATCCCAG	4	0.54	None
OT_Cdkn2a-ex1ß_5	Chr16	1	39487042	AAGTGAAGTTTGTGCGTTCCCAG	4	0.34	None
OT_Cdkn2a-ex1ß_6	Chr13	-1	50417915	TGCTGCAGTTCGTGCGGGCCAAG	4	0.07	NM_175401
OT_Cdkn2a-ex1ß_7	Chr11	-1	120809527	TGTGGAAGTTCGTCAGATCCTGG	4	0.06	NM_007988
OT_Cdkn2a-ex1ß_8	ChrX	1	58919170	TGGTGAAGTTTCTGAGCTCCAAG	4	0.04	NM_023774
Cdkn2a-ex2							
OT_Cdkn2a-ex2_1	Chr4	1	148874074	GTGGGAGATCTGCGTTCCGTAAG	4	0.42	None
OT_Cdkn2a-ex2_2	Chr7	1	126924957	GTGCGTTCTTTGCGTTGCGTGGG	4	0.15	None
OT_Cdkn2a-ex2_3	Chr4	1	45255256	GTGCCATATTCCAGTTCCGCAAG	4	0.15	None
OT_Cdkn2a-ex2_4	Chr2	1	180925924	GTGGGACATTTGGGTTCCTCTGG	4	0.13	None
OT_Cdkn2a-ex2_5	Chr8	-1	72157092	GTTCAATATTTGTGTTCTGCCAG	4	0.12	None
OT_Cdkn2a-ex2_6	Chr11	-1	95675148	GTGTGATATTGACGTTCTGCAAG	4	0.08	NM_008831
OT_Cdkn2a-ex2_7	Chr3	-1	53474746	GTGCGATAGTTGCATGCGGCCGG	4	0.00	NM_173382
Cdkn2b							
OT_Cdkn2b_1	Chr11	1	116110179	ACCGGCTCCCGGAGCGGTTCCAG	4	0.73	NM_172570
OT_Cdkn2b_2	Chr17	-1	25067936	GGGGCCTCCTGAAGGGGTTCTGG	3	0.72	None
OT_Cdkn2b_3	Chr5	-1	112046540	GGCGGCTGCAGAAGCGGTTGAGG	4	0.61	None
OT_Cdkn2b_4	Chr14	1	21750555	CCCGCCTCCCGAAGCAGTTCCGG	3	0.56	NM_026283
OT_Cdkn2b_5	Chr14	1	25710848	GGATGCTCCCGAAGCGGTGCTGG	4	0.50	None
OT_Cdkn2b_6	Chr2	-1	148044131	TGCGCCGCCAGAAGCGCTTCAAG	4	0.39	NM_010446
Pten							
OT_Pten_1	Chr1	-1	96400219	CCTATCGATTTCTTTGATGATGG	3	2.53	None
OT_Pten_2	Chr10	1	11786839	AATACCGGTCTCTTTGATGATGG	4	1.39	None
OT_Pten_3	Chr6	1	110140664	TGTCACGATGTCTTTGATGAAGG	4	1.32	None
OT_Pten_4	Chr1	-1	146699106	GCTTACGATGTATTTGATGATGG	3	1.21	None
OT_Pten_5	Chr12	-1	8410402	AGTAGCTATCTCTTTGATGAGAG	4	0.93	None
OT_Pten_6	Chr2	1	37427652	TGTAACAATGTCTTTGATGAAAG	4	0.90	NM_146253
OT_Pten_7	Chr3	1	138451237	GCTGACACTGTCTTTGATGATAG	4	0.86	NM_007410
OT_Pten_8	Chr10	-1	63017546	GGAAACGATGGCTTTGATGACAG	4	0.72	NM_001079824
Rosa26.1							
OT_Rosa26.1_1	Chr16	1	19408733	TTGATTATTTGATACCATATAAG	2	2.47	None
OT_Rosa26.1_2	Chr8	-1	55887271	TTGTTTAATGTCTACCATATGGG	3	1.45	None
OT_Rosa26.1_3	Chr4	-1	93809596	TTGATTAATTTCTACCATATTGG	3	1.21	None
OT_Rosa26.1_4	Chr10	1	37034194	ATGTTTCTTTGCTACCATATTAG	4	0.90	None
OT_Rosa26.1_5	Chr6	-1	38705196	CTTCTTATTGACTACCATATCAG	4	0.80	None
OT_Rosa26.1_6	ChrX	1	155324145	TTGATTGTTGTATACCATATCAG	3	0.46	NM_016764

OT_Rosa26.1_8	Chr17	-1	85607421	CTGATTATTACCTGCCATATCGG	4	0.12	NR_015387
Rosa26.2							
OT_Rosa26.2_1	Chr13	-1	54604762	TGGACCCAGAAGACTATCTGTAG	3	1.59	None
OT_Rosa26.2_2	Chr2	-1	42198522	TAGGACCAAAAGACTATCTGTAG	3	1.52	None
OT_Rosa26.2_3	Chr3	-1	95594328	TAATTCCACAAGACTATCTCAAG	3	1.42	None
OT_Rosa26.2_4	Chr10	-1	70183548	TTGTCCCTCAAGTCTATCTGCAG	3	1.12	None
OT_Rosa26.2_5	ChrX	-1	51672728	TGCTCCCTGAAGACTATCTGTGG	4	0.84	None
OT_Rosa26.2_6	Chr3	1	96637363	TTATCCTACAAGACTATGTGTAG	4	0.21	NM_011069
OT_Rosa26.2_7	Chr11	-1	96447670	AAGTCCCCCATGACTTTCTGCAG	4	0.15	NR_033524
OT_Rosa26.2_8	Chr4	-1	117897880	CAGTCCCACATAACTATATGAGG	4	0.09	NM_146152
Smad4							
OT_Smad4_1	Chr18	1	39700412	AATAGCAGCTCATAGTGATAGAG	4	0.91	None
OT_Smad4_2	Chr6	-1	127223185	ATAACCCGCTTATAGTGATGTGG	3	0.83	None
OT_Smad4_3	Chr8	-1	85849958	ACAGCCCTTACATAGTGATAGGG	4	0.77	None
OT_Smad4_4	Chr13	1	31135739	ACAAACATCTCCTAGTGATATGG	4	0.47	None
OT_Smad4_5	Chr1	1	168232264	GAAACCAGCTCAAAGTGATAGAG	4	0.40	None
OT_Smad4_6	Chr10	1	21148552	ACTATCTGCTCAAAGTGATACGG	4	0.38	NM_001198914
Trp53							
OT_Trp53_1	Chr17	-1	54419858	AACACTTGGAGGGCTTCACTTGG	2	4.57	None
OT_Trp53_2	Chr5	-1	106714108	ATCACTTGGAGGGCTTCACTCAG	3	1.74	None
OT_Trp53_3	Chr10	-1	109647934	GGCTGTCAGAGGGCTTCACTCAG	4	1.36	None
OT_Trp53_4	Chr9	-1	49800050	GTCTGTCAGAGGGCTTCACTGAG	4	1.36	None
OT_Trp53_5	Chr5	-1	117024931	CACACTGGGAAGGCTTCACTTAG	3	1.07	None
OT_Trp53_6	Chr2	-1	35730631	GGCAGTCAGAGGTCTTCACTCAG	4	0.57	NM_001114125
OT_Trp53_7	Chr2	-1	62501156	GACAGTCTGAAGGCTTCACATGG	4	0.37	NM_007986
OT_Trp53_8	Chr2	-1	158261400	AACACTCGGAGGCCATCACTGGG	3	0.33	NM_177850

Supplementary Table 3 | Genomic regions analysed for off-target effects by CRISPR/Cas9 *in vivo* electroporation. The respective chromosome, location, genomic sequence (off-target binding site) and the number of mismatches (in regard to the on-target sequence) of each off-target are given. The top five off-target regions for each sgRNA plus the three best-ranked exonic regions have been analysed. The off-target score indicates the strength of the guide-DNA interaction and is computed by taking into account the total number, the pair-wise distribution and the proximity of mismatches to the PAM site[14]. The lower the score the less likely a Cas9-mediated mutation will occur.

Name	Forward primer	Reverse primer	T_a [°C]
Apc			
OT_Apc_1	CTGAGTGTGGTGCTATACTCAAG	ACTAGGATTAGGACCTAGGAAACA	60
OT_Apc_2	AGATCTGCAGTTCACCCCAA	GGGAGTCCAGGAAGCAGAAT	60
OT_Apc_3	AGTTACTGGTGGCTGTAAGACA	AGAGTGGCAGTTCAAGGTAGT	60
OT_Apc_4	ATCCAACGCTGATTCCTTGC	GGGAGGTGATTGAGAGGGAC	60
OT_Apc_5	CCTGGTTTTACGTTGCTGCT	CTATTTGCCTGCACCTCCAG	60
OT_Apc_6	CAATGCAAAAGGTGTTCTGACA	TCACCACCCTTGCTGTAACT	60
OT_Apc_7	CACTTGCTTCAGTCTGAGCC	CCTGCAGTCAACCTTGGTTC	60
OT_Apc_8	CGAACCTGTCAGTTGCAAGT	TGCGATGTTCTGGGCTATCT	60
Arid1a			
OT_Arid1a_1	TCCAGATGCCAACCCCTATC	GCCACAGACCCTATTCCTCA	60
OT_Arid1a_2	TGAGAGGGTCACGAGTTGG	CTATTGCCCCAGACCCAGAG	60
OT_Arid1a_3	TGTCTACGATCACAGTGCAGT	ACACAGGCTGTAACTCTGAAGA	60
OT_Arid1a_4	CAGAGGAAGTTGGGTGAGGA	TCATGCTCATCAGGGCTTCT	60
OT_Arid1a_5	GCCAACAGGTGAGTCTTCTAAC	CAGGCCCATGTTGTCTGAAG	60
OT_Arid1a_6	CGGCAAGTTCTGTTTGTGCT	GTCTGGGTCTCATCTCCTGG	60
OT_Arid1a_7	TCCTCGAAGTAGACATATCCACA	TGCAAAGGTTCTTCTGGAGC	60
Arid1b			
OT_Arid1b_1	GGCTCTAAAACCCCCAACCT	GTTCCCAACTGGGAGCCTGA	60
OT_Arid1b_2	GTGGTAGGAGCTTGGAGTCG	AACCAGGGGATCAAGGACTG	60
OT_Arid1b_3	TGCTACATCTGGAGCCTCTTG	CACCTTCAGAGAGCAGCAGTA	60
OT_Arid1b_4	TGAAAAGAGAACGACGGCGA	ACTCCTCTACCTGTGTCCCG	60
OT_Arid1b_5	CATCCCAGACCCCTTTCAGC	GAGTGGGCAGGAGAAATCCG	60
OT_Arid1b_6	GTGGTGTGTCTGATTGCCTT	GCGCAGCACTCCTGTAAGA	60
Arid5b			
OT_Arid5b_1	CACAACCAAAGGTCTGACGC	AAGCTGAAAGTCCCAGTTGGAT	60
OT_Arid5b_2	ACCAGTTGGTTTGTGCAGGG	TGGTAGCTGGAACCTTTCCC	60
OT_Arid5b_3	GAAAAGCCCAAACAAGCTGGA	TGTGCCATTTGCCCAGGTTT	60
OT_Arid5b_4	GCCAGCTAAACTTATACTCTGGTTG	CTGCACCATGCCTGCCTAT	63
OT_Arid5b_5	GATTTCTGTGGACCAGCACC	TCTGTGCACTTTGGCGCTTA	63
OT_Arid5b_6	ATTGCTTTGCTCTCCCCAGG	GTACCTCAGGCATCCCACAT	60
OT_Arid5b_7	TGGTTGACCTATGCACGACC	GCACATTGCAAAGGGTCTCA	60
Atm			
OT_Atm_1	TGAGGGTTCAAACCCACCCC	AGAAGATCAGTGGTCAGCCC	63
OT_Atm_2	ACTAGCCCCCTTTTGCTCTT	GGAGCTCAGGAACTAGGGGT	60
OT_Atm_3	CTGGAGCAGCGGCTTTAAGT	CTGCTGTCCCCACATGGAAG	60
OT_Atm_4	ACCTCGAGAATTGCAGGTCC	GGGAATTGAGAAAGTGTTGGAGT	60
OT_Atm_5	GGCCAGTCATCTATGCAGTCA	TCTTTATTCCAGCCGACGCT	60
OT_Atm_6	TGTGATACACGGGACTGTGAG	CATGCCATGCCATTTGTGC	60
OT_Atm_7	CCTGTGAGTGCTGAGGTTCA	CAGCGAAAGCTGAAACCGTA	60
OT_Atm_8	TCGGGTGCTCTTAATCTGCTG	TGTTCCTTGGAGTCCTTGTTGA	63
Brca1			
OT_Brca1_1	GACTTCGTGGACAGAATGGC	TCCAGCCCTGTTTGATTCCT	60
OT_Brca1_2	GAGAACTGCAGAGCCCATTG	ACCGACATTTTCCCCTCCTT	60
OT_Brca1_3	TCCAAAGGCTGCTAGTGGAA	CCTCGACCCCTCCCAATTTT	60
OT_Brca1_4	CCCAACACAGCCCACTACA	ACCTGCAGAGTAAAGGGCTC	60
OT_Brca1_5	TGGATTCCAGCCTCTGTCAA	TGTCCCTAGCCAGTACCTCT	60
OT_Brca1_6	TAGCAGGGACCTCAAAGTGG	ATAGCAGCCCATGAAGCCAG	63

OT_Brca1_7	GCACTGTAAGCTCAACCCAG	CCTCTGCCACATGAGTACCA	60
OT_Brca1_8	ACATGACTGGAGTTAGAAAAGGA	TGTGCTTGCTATTCCTATGATGA	60
Brca2			
OT_Brca2_1	CACAGTAGGTTGGGTCTTCC	GACAGGGTTGGAGAGTGCC	60
OT_Brca2_2	GCGCTGTTATTTCCTCCGTT	AGCAAGGCCAGTGATCTCAT	60
OT_Brca2_3	TGAGCAAGTCACTTTGGAAAACA	AAGTGGGAACTTCAGGAGGG	60
OT_Brca2_4	CACTGAGTGTCATGCTTGGC	ACTAGTGAGCCCTGCCTTTC	60
OT_Brca2_5	GACACAGGAAGAGGGAGACA	ATCAAGCCACCAGAATCCCT	60
OT_Brca2_6	TGCATTTCCTTTGACACCAGT	ATCAGAGATCTCCGTGGCTG	60
OT_Brca2_7	AGAAGGAATTTGGGATTTTGGCA	TGGAGAGTGAGCTAGCCAAG	60
Cdkn2a-ex1ß			
OT_Cdkn2a-ex1ß_1	GCTTCCCTGAAACCTGCATC	CATCAAGGACTAGGAGCAATGA	60
OT_Cdkn2a-ex1ß_2	GTTGCCCTCATCTCAGACCT	TTCCAAGTGCAGCAAAGGTC	63
OT_Cdkn2a-ex1ß_4	GGGGAGAGGGTCTAGAAGGA	TCCACAGATCATTGGCGAGA	60
OT_Cdkn2a-ex1ß_5	GGCATCTTTTCATTTGTCAGCC	ACACAGACACACAGATCCAAT	60
OT_Cdkn2a-ex1ß_6	ACTTCAGTGATCGCTAGGCC	CACACAGTGGGGCATAGAGA	60
OT_Cdkn2a-ex1ß_7	TGAGGACATGCACACAGACT	AATGCTTGGCTGGGTGATTG	60
OT_Cdkn2a-ex1ß_8	CTGCAGAGAGTTCCCAGGAA	CTCTTCATTGCTGATCCGCC	60
Cdkn2a-ex2			
OT_Cdkn2a-ex2_1	TGGGCTTGTTTTAAAGGGGC	CAATGTCTGCTGCTCACCTG	63
OT_Cdkn2a-ex2_2	GTCTGTTTGGATGCCCTTGG	AGGCTACTCTTGCTGTCTCC	63
OT_Cdkn2a-ex2_3	AAACTGAACTTGCTCGGCTC	TTGAGCATGAGAGGGAAGCA	60
OT_Cdkn2a-ex2_4	TACCACTTCCTTCCCTGCAG	ATTGACTGTCCTACCCTGGG	60
OT_Cdkn2a-ex2_5	TTACCTAACTCCTGGGGCAG	CAGGAAGCTAGACTGTGCCT	60
OT_Cdkn2a-ex2_6	CCATCCTGTCAGTGGTTCCT	GCTACCTACCCACCACTACTC	60
OT_Cdkn2a-ex2_7	ACTGGGGCATCTTCAGTCTC	AGTGAAAAGCCCCAATGATAAGT	60
Cdkn2b			
OT_Cdkn2b_1	GACAGCGTGTGGTTCTTGC	GAAGTCTCAAGAGGGTCGTGC	63
OT_Cdkn2b_2	TTTTGGGCAAAACAGACCCG	TCAACCTGATTGCTGCCTTTC	60
OT_Cdkn2b_3	TCATCCACCTCTCCCTTCTCT	TGTGTAGATGCTCTGGGGAC	63
OT_Cdkn2b_4	AGGACTGACTGAGAAGGGGTT	CTAAGTCGCCGTTGGAGTCG	60
OT_Cdkn2b_5	GATACGGGTCTTGCTTCTGGA	GCCCCTTGAGCATCACTTGG	60
OT_Cdkn2b_6	GAGGCAGGTGCTCCCTTTAG	CCTCTTCCCTTTCTACCGGC	60
Pten			
OT_Pten_1	CAAGAGAAAGACAAGGCATGGT	AGAAGGGAGGAGGGAAGGAA	60
OT_Pten_2	GGAGCAGCTTGGAGTCTGAT	CATTGCCAGCACAGTTCTCA	63
OT_Pten_3	GGAACATTAAGAGTGAAACAGCT	AAATAGGTGGCAGAACGGGT	63
OT_Pten_4	CATGCAACAACAGAGGACACA	TCCTTCTTCTGACCAAATGTGA	63
OT_Pten_5	AACAATGCTCAGAGGGTCCC	GATGGAATGTTGGGCCTCAA	60
OT_Pten_6	AAGGGTGGACTACAAAAGAGC	ACAGAAAGGTTTGTCTTGGCC	60
OT_Pten_7	GCTGTGGTATTTCAACTGGCT	TGACCTTCACGTTGCCAATG	63
OT_Pten_8	CCATAGCCATGTCCTCCCAT	GCTGCAAACATTAATGAAGAAGC	60
Rosa26.1			
OT_Rosa26.1_1	CCCATGTGTAGTGTCAATCATCC	TCACACAGGTAAAGTAATGTGTTGT	60
OT_Rosa26.1_2	CTGGGGCATCACCAAATTGT	CGGCTGAACTTAATTGCTCCTTT	60
OT_Rosa26.1_3	AAAGCCTGGAGTTTCTGTTGCT	TTTACTAGCTCAGAGATTGGGACT	60
OT_Rosa26.1_4	AACCCAGGGACTGAAAAGCA	AGGGCTTTTGTGCATGCTAAG	60
OT_Rosa26.1_5	CATTCCTGAGGTGGCCAGG	AAAGGCTTCGAACCAAAAATGT	60
OT_Rosa26.1_6	TTACAATGGCAAACGAATATGGT	AGCAGACTCGGTGCTCTTAAC	60
OT_Rosa26.1_8	CCTCAATCCCACTCTACGTTT	AGGGGTGTATTTGCAACTCGT	60

Rosa26.2			
OT_Rosa26.2_1	CTGTCCCCCAGTAGCCAAAG	CTCTGCTATCACAGTGCCCC	60
OT_Rosa26.2_2	TCATAACTGAGATTGTTAAAGACCA	CAGGGTTGGGACACTAGTTGA	60
OT_Rosa26.2_3	GAAATGACATTCCCCAGCCC	TTGGTCCTCAGAACCCTCAG	60
OT_Rosa26.2_4	TGCTTGCCTTCATCTGACCC	TCACGGTAGTGCTGGCTAGA	60
OT_Rosa26.2_5	AGACTTCCATGGGATGTGAGAAAT	AATTTCATTCGAAGGGTGTATCTC	60
OT_Rosa26.2_6	CTGGGGAACTCAACAGATGC	TTGATTCCCAGAGCCCACAT	60
OT_Rosa26.2_7	TATTCTGGGGTGCGTTCAGC	CATGCCAGGCGTTTGTTAGC	60
OT_Rosa26.2_8	AGGCAAAGGGGCACTAAAGG	GTCCTTCACTCTGCCGTCTC	63
Smad4			
OT_Smad4_1	CATCATCTCCAAGGCCCTCA	GCCATTCCAGGGATCAAACC	60
OT_Smad4_2	CAGATATGGTGGTGCATGCC	TTGGAAAGCAGAGCAACAGG	60
OT_Smad4_3	GGGGTTCCTGGGAGTCTTTT	TACTGTGGCCTTGAGAAGCA	60
OT_Smad4_4	TAAGCAGCACTCACCACCAA	GCTCAGTCACCTAAGCTTGT	60
OT_Smad4_5	AAAGTGGGACTCATAGGGCC	TCCCGTCTCAGGTCACAAAA	60
OT_Smad4_6	TAATGCCTGCTGTCCCTTCA	TGAGATCATCTGACGGGCAA	60
Trp53			
OT_Trp53_1	CCTAGCATTCAGGCCCTCAT	TGAGGGGAGGAGAGTACAGT	60
OT_Trp53_2	GGATTGTCCCTTGTACCACTTC	AACAAATGTGCGGGCAACTT	60
OT_Trp53_3	GCATGCACTGAACAGAAATTGG	TCAGAGGAGATTTGCTTGGGA	60
OT_Trp53_4	CCCTGGCTCTTCTGTGTGTA	GAACCCGCAGCATGTGATAG	60
OT_Trp53_5	CATGATGCCTGTTCACGAGG	CTGGTAAAAGGTGCTGGCTT	60
OT_Trp53_6	CATGCTGTTTGGGTGGAAGG	AGAAAAGAGGGGCTGGTTCC	60
OT_Trp53_7	CTACCCGGCAATGAACAGGT	CCAAGTGGCCAAGAAGCAAA	60
OT_Trp53_8	GGCTTGCCGTCTTTGTTGAT	AAGTGGACAGTTCTCCCAGC	60

Supplementary Table 4 | PCR primer used for the off-target analysis. Primers used for amplifying CRISPR/Cas9 off-target sites and annealing temperature are given. The length of PCR products ranges from 400bp to 450bp.

Gene	Ensembl ID	CCDS	sgRNA sequence	Exon
Apc	Apc-001 (ENSMUST00000079362)	CCDS29125	TCAGTTGTTAAAGCAAGTTG (AGG)	2
Arid1a	Arid1a-201 (ENSMUST00000105897)	CCDS38908	TTAGTCCCACCATACGGCTG (AGG)	2
Arid1b	Arid1b-201 (ENSMUST00000115797)	CCDS49929	CTGTGCACCTGGGGGACCGT (AGG)	2
Arid5b	Arid5b-201 (ENSMUST00000020106)	CCDS35929	GCTATGCAAATCGGATCCTT (TGG)	2
Atm	Atm-001 (ENSMUST00000118282)	CCDS40636	GGCTGTCAACTTCCGAAAAC (GGG)	7
Brca1	Brca1-001 (ENSMUST00000017290)	CCDS25474	AAATCTTAGAGTGTCCGATC (TGG)	2
Brca2	Brca2-201 (ENSMUST00000044620)	CCDS39411	TAGGACCGATAAGCCTCAAT (TGG)	3
Cdkn2a-ex1β	Cdkn2a-201 (ENSMUST00000107131)	CCDS18350	TGGTGAAGTTCGTGCGATCC (CGG)	1
Cdkn2a-ex2	Cdkn2a-001 (ENSMUST00000060501)	CCDS38812	GTGCGATATTTGCGTTCCGC (TGG)	2
	Cdkn2a-201 (ENSMUST00000107131)	CCDS18350		
Cdkn2b	Cdkn2b-201 (ENSMUST00000097981)	CCDS18351	GGCGCCTCCCGAAGCGGTTC (AGG)	1
Pten	Pten-001 (ENSMUST00000013807)	CCDS29753	GCTAACGATCTCTTTGATGA (TGG)	1
Rosa26.1	Gt(ROSA)26Sor	OTTMUSG00000034748	ATATGGTAGCCAATAATCAA (TGG)	intergenic
Rosa26.2	Gt(ROSA)26Sor	OTTMUSG00000034748	TAGTCCCACAAGACTATCTG (AGG)	intergenic
Smad4	Smad4-001 (ENSMUST00000025393)	CCDS29337	GACAACCCGCTCATAGTGATA (TGG)	2
Trp53	Trp53-202 (ENSMUST00000171247)	CCDS48826	GACACTCGGAGGGCTTCACT (TGG)	3

Supplementary Table 5 | sgRNA target regions by in vivo electroporation of the murine pancreas. For all respective genes, Ensembl IDs and consensus coding sequences (CCDS) are shown. The used sgRNA sequence including PAM sequence and the targeted exon are displayed.

Gene	Forward Primer	Reverse Primer
Apc	GCGAATAAGCACCACTCCTC	AAGAATGAACCAACACCAAGG
Arid1a	GTTCTGATTCCTGTGCTCGC	TCCATCACCTACCTGCTGTG
Arid1b	AGTTCTGGGGTACTTGGAATCA	GGTACTGCAAGCCTCCCA
Arid5b	TGGCTTGCACGGACCTTATA	ATCAGCAGTTGGACGGTCTT
Atm	TCCTTTTCAACTGTTCCTGTTACA	GACAATGGAAAGGCGAGTCA
Brca1	AGCGTGAGAACTCCTCCAAA	CTGCCATGAGGAAGAACACA
Brca2	TCACGAGTTTCTCCGTGTCA	GCTCTGGCTGTCTCGAACTT
Cdkn2a-ex1β	TCTCACCTCGCTTGTCACAG	AAGTACTCCATCTCCCGGGA
Cdkn2a-ex2	TCAACTACGGTGCAGATTCG	CGGGTGGGTAAAATGGGAAC
Cdkn2b	CCGAAGCTACTGGGTCTCC	CACTTGCCCAGCTTGTACG
Pten	TGCGAGGATTATCCGTCTTC	CATCCGTCTACTCCCACGTT
Rosa26.1	TCTGATGCCCTCTTCTGGTG	GGCTAAACTCTGGCCCTACA
Rosa26.2	GGAAGGATTGTCTGTGCCCT	ATTTTCCAAAGCCCTCCCCA
Smad4	TGCAGTGTCACAGATGCTCA	CTCAGGAACTGGAGGAAGCA
Trp53	ACATAGCAAGTTGGAGGCCA	CCACTCACCGTGCACATAAC
**Library barcoding primers**		
PE adapter top strand	ACACTCTTTCCCTACACGACGCTCTTCCGATCT	
PE adapter bottom strand	GATCGGAAGAGCGGTTCAGCAGGAATGCCGAG	
PE 1.0	AATGATACGGCGACCACCGAGATCTACACTCTTTCCCTACACGACGCTCTTCCGATCT	
iPCRtag	CAAGCAGAAGACGGCATACGAGATXXXXXXXXCGGTCTCGGCATTCCTGCTGAACCGCTCTTCCGATCT	

Supplementary Table 6 | Primer sets for amplification of genomic target regions for Sanger and Next Generation sequencing. Indicated primer sets produce amplicons between 400 and 600bp in length. Oligos used for Ilumina next generation sequencing are stated beneath.

Antibody	Vendor	Host	Pre-treatment	Dilution
E-cadherin	Cell signaling	rabbit	10 mM sodium citrate buffer pH 6.0; 95°C; 10 min	1:400
Cytokeratin 19	Hybridoma bank (TROMAIIIc)	rat	EDTA; 100°C; 20 min	1:500
Cleaved Caspase 3	Cell signaling	rabbit	10 mM sodium citrate buffer pH 6.0; 100°C; 20 min	1:200
GFP	Fitzgerald	rabbit	EDTA; 100°C; 20 min	1:200

Supplementary Table 7 | List of primary antibodies used for IHC.

Primer	Sequence
EGFP-tdEG-LP	CCATGTGATCGCGCTTCTCGT
caggs-sc-UP4	GTTCGGCTTCTGGCGTGT
tdEG-nest-INf	GCAACGTGCTGGTTATTGTG
tdEG-nest-INr	CTCGATGTTGTGGCGGATCT

Supplementary Table 8 | Nested PCR primers used for detection of recombined *R26*$^{mT\text{-}mG}$ allele after somatic PGK-Cre delivery by *in vivo* electroporation of the murine pancreas.

Supplementary References

1. Jiao Y, *et al.* Whole-exome sequencing of pancreatic neoplasms with acinar differentiation. *The Journal of pathology* **232**, 428-435 (2014).

2. Furlan D, *et al.* APC alterations are frequently involved in the pathogenesis of acinar cell carcinoma of the pancreas, mainly through gene loss and promoter hypermethylation. *Virchows Archiv : an international journal of pathology* **464**, 553-564 (2014).

3. Biankin AV, *et al.* Pancreatic cancer genomes reveal aberrations in axon guidance pathway genes. *Nature* **491**, 399-405 (2012).

4. Jones S, *et al.* Core signaling pathways in human pancreatic cancers revealed by global genomic analyses. *Science* **321**, 1801-1806 (2008).

5. Shain AH, *et al.* Convergent structural alterations define SWItch/Sucrose NonFermentable (SWI/SNF) chromatin remodeler as a central tumor suppressive complex in pancreatic cancer. *Proceedings of the National Academy of Sciences of the United States of America* **109**, E252-259 (2012).

6. Lynch HT, Deters CA, Lynch JF, Brand RE. Familial pancreatic carcinoma in Jews. *Familial cancer* **3**, 233-240 (2004).

7. Goggins M, *et al.* Germline BRCA2 gene mutations in patients with apparently sporadic pancreatic carcinomas. *Cancer research* **56**, 5360-5364 (1996).

8. Ozcelik H, *et al.* Germline BRCA2 6174delT mutations in Ashkenazi Jewish pancreatic cancer patients. *Nature genetics* **16**, 17-18 (1997).

9. Caldas C, *et al.* Frequent somatic mutations and homozygous deletions of the p16 (MTS1) gene in pancreatic adenocarcinoma. *Nature genetics* **8**, 27-32 (1994).

10. Schutte M, *et al.* Abrogation of the Rb/p16 tumor-suppressive pathway in virtually all pancreatic carcinomas. *Cancer research* **57**, 3126-3130 (1997).

11. Redston MS, *et al.* p53 mutations in pancreatic carcinoma and evidence of common involvement of homocopolymer tracts in DNA microdeletions. *Cancer research* **54**, 3025-3033 (1994).

12. Asano T, Yao Y, Zhu J, Li D, Abbruzzese JL, Reddy SA. The PI 3-kinase/Akt signaling pathway is activated due to aberrant Pten expression and targets transcription factors NF-kappaB and c-Myc in pancreatic cancer cells. *Oncogene* **23**, 8571-8580 (2004).

13. Hahn SA, *et al.* DPC4, a candidate tumor suppressor gene at human chromosome 18q21.1. *Science* **271**, 350-353 (1996).

14. Hsu PD, *et al.* DNA targeting specificity of RNA-guided Cas9 nucleases. *Nature biotechnology* **31**, 827-832 (2013).
